# Supplementary material for: A genome-wide association study identifies six novel risk loci for primary biliary cholangitis
Source: Nat Commun. 2017 Apr 20;8:14828. doi: 10.1038/ncomms14828 (PMC5429142; doi:10.1038/ncomms14828)
Supplement: Supplementary Information — Supplementary Figures and Supplementary Tables [file ncomms14828-s1.pdf]

Supplementary information

1. Supplementary Figures

Supplementary Figure 1: Geographic distribution of healthy control and PBC samples included in the study

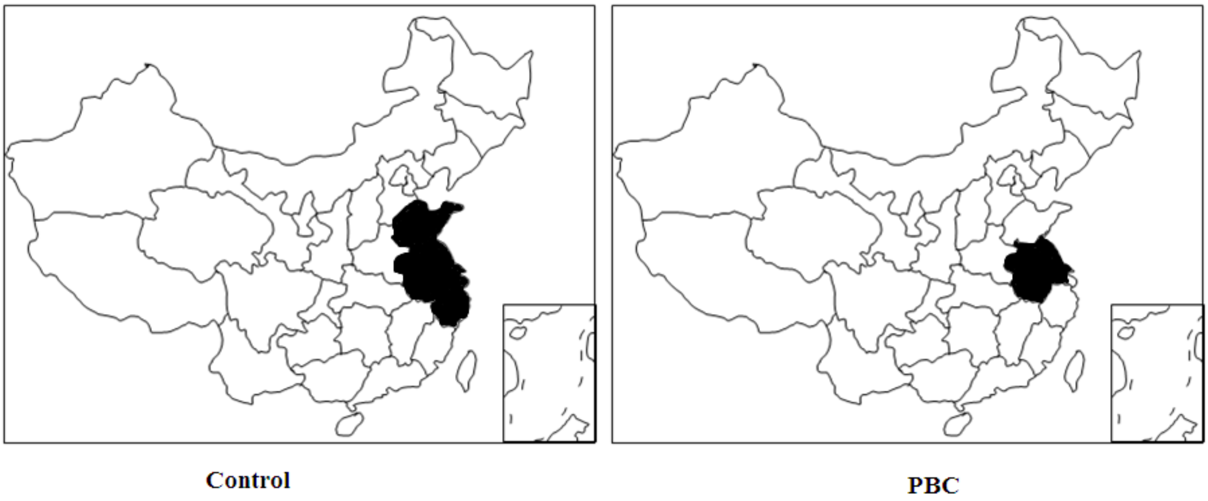

Supplementary Figure 2: Principal component analysis of PBC cases and controls.

The principal component analysis (PCA) was performed in 5,199 samples (1,125 cases, 4,074 controls) in Illumina HumanOmniZhongHua-8 Array (v1.1). PC, principal component.

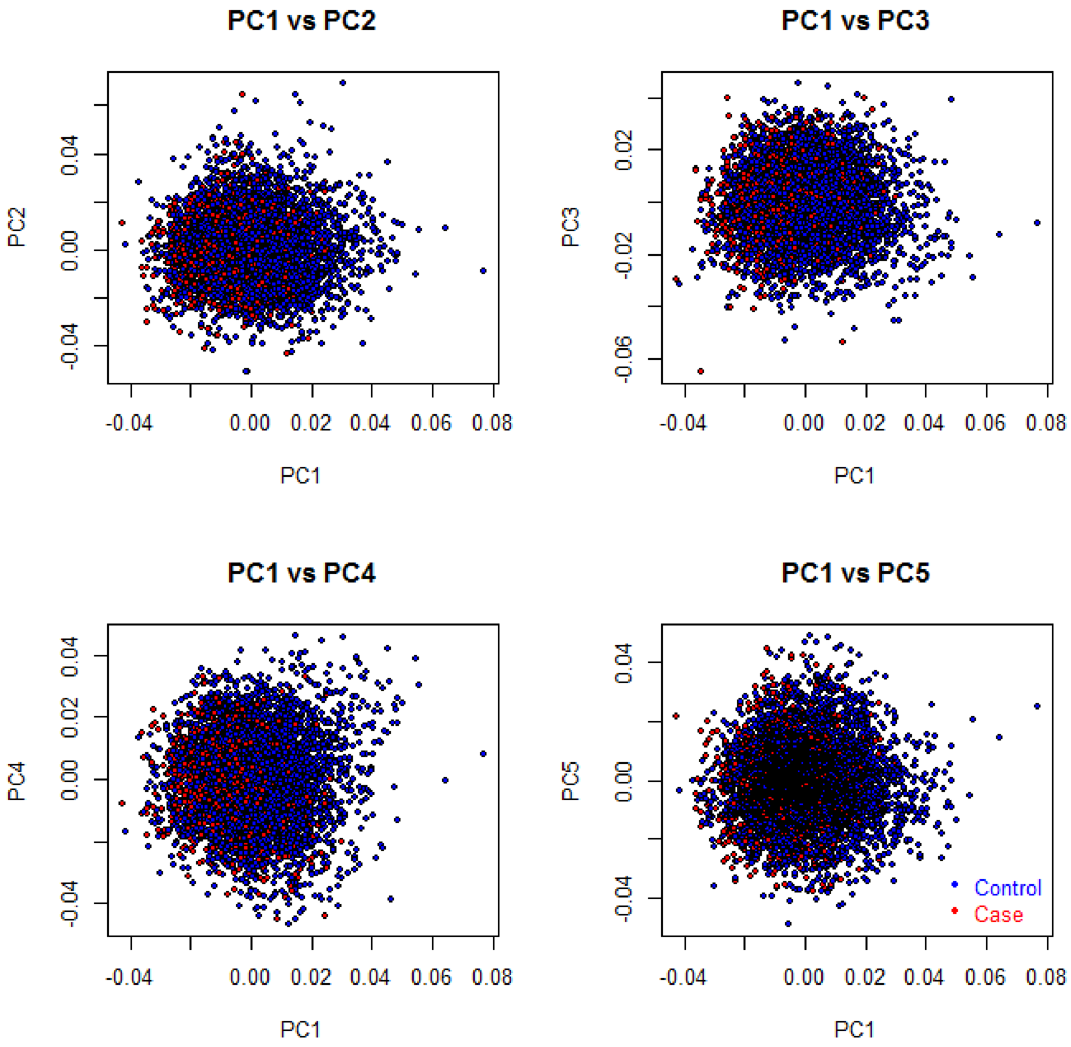

Supplementary Figure 3: Quantile-Quantile (Q-Q) plots of PBC GWAS data.
The red line is for null expectation and the plot in black is for the P values from all the 796,160 SNPs, whereas the plot in blue is for P values for SNPs excluding the 10,790 SNPs within the MHC region (chr. 6: 26–34 Mb).

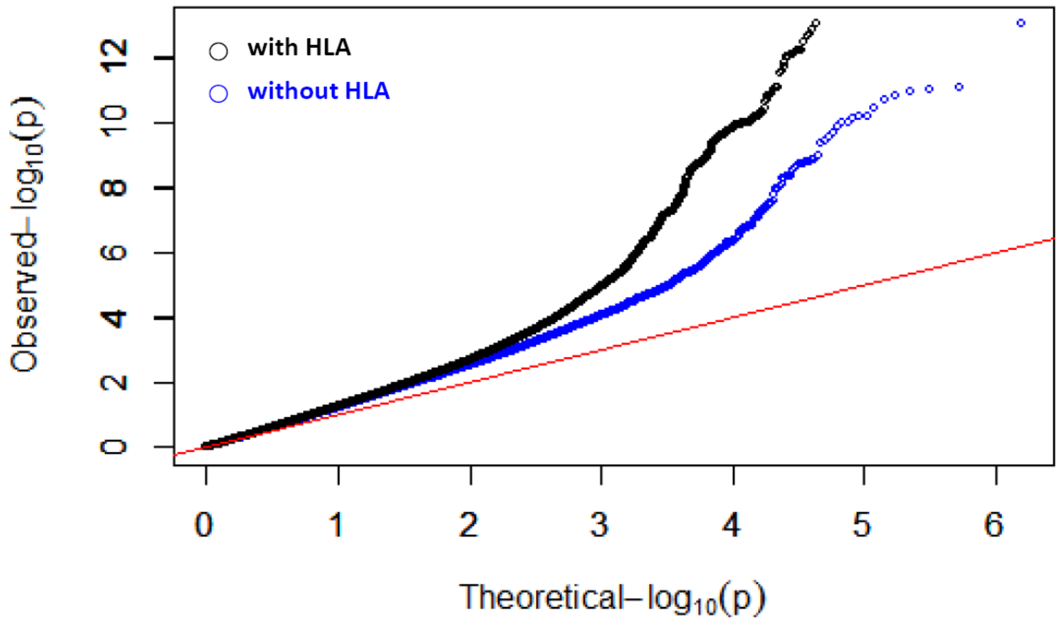

Supplementary Figure 4: Locus specific plots for the known significant loci identified in GWAS.

a. TNFSF15 locus

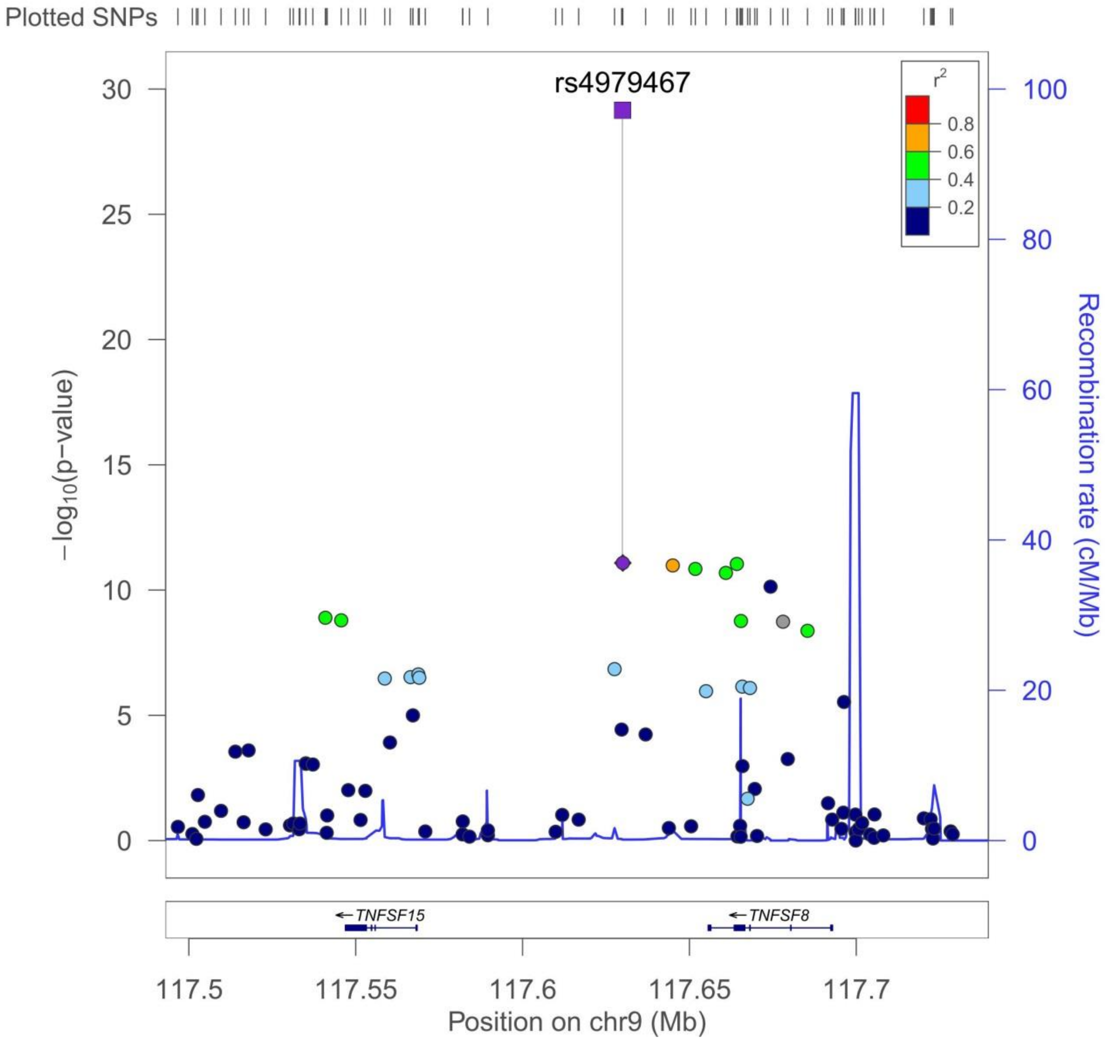

b. 17q12 locus

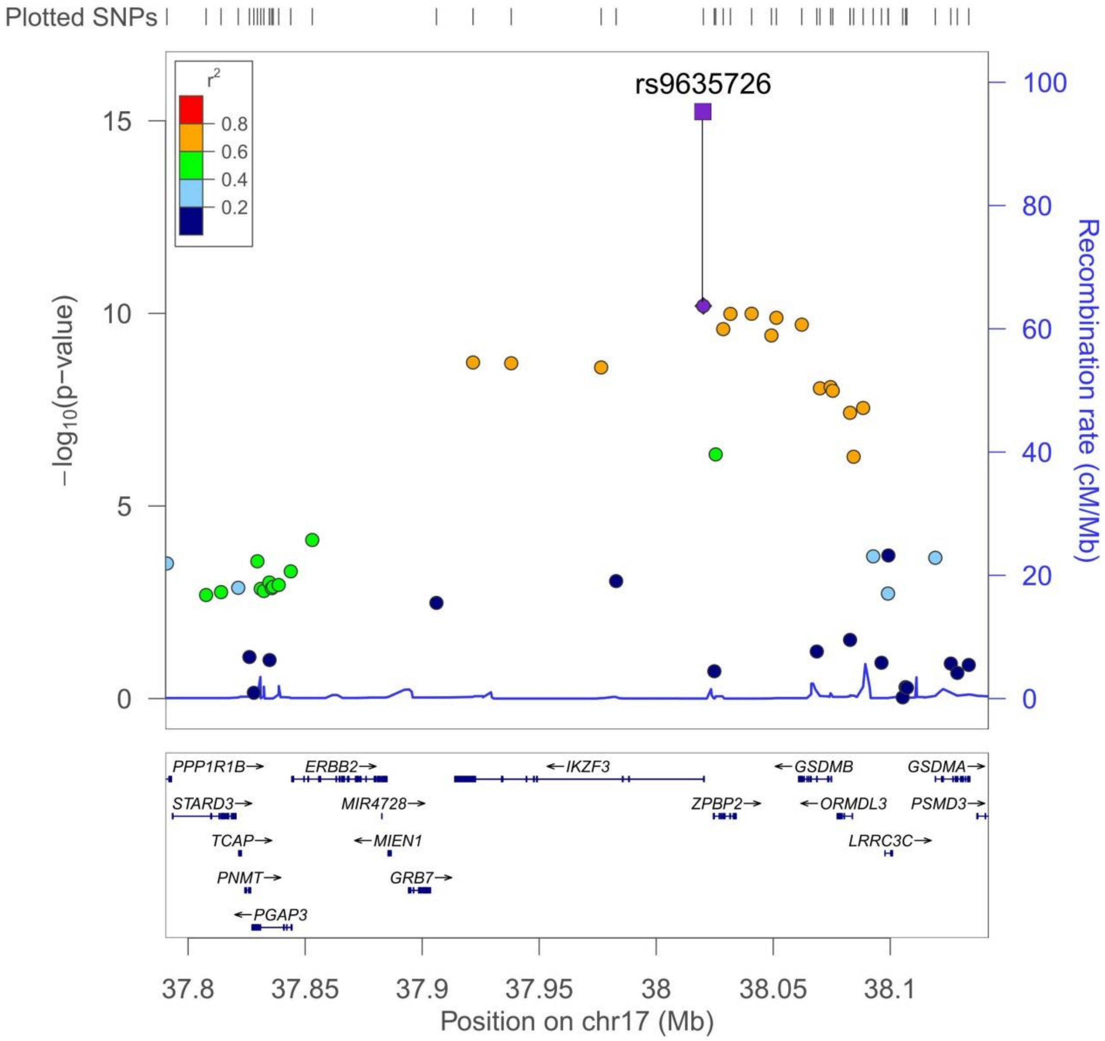

c. **DDX6-CXCR5 locus**

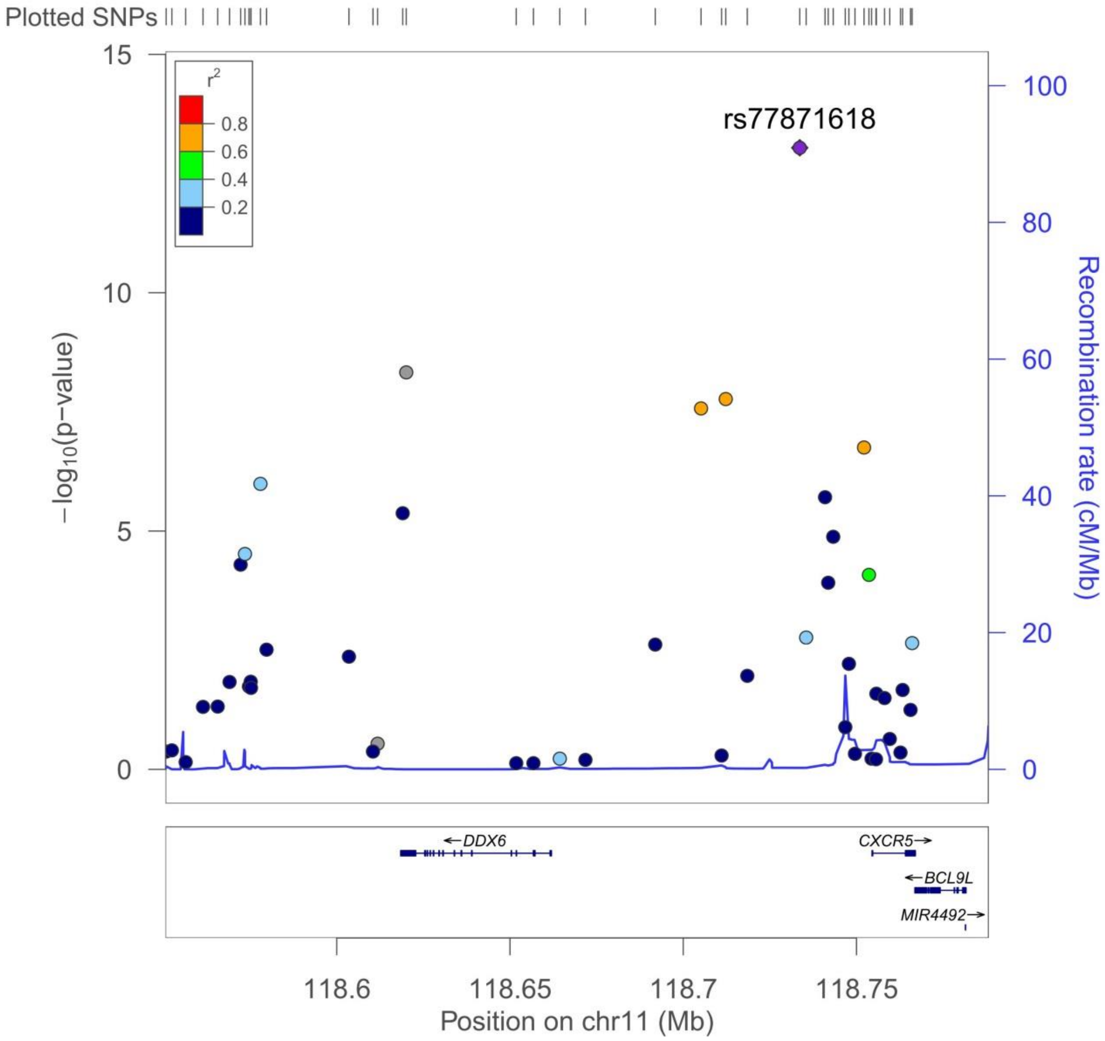

d. CD80 locus

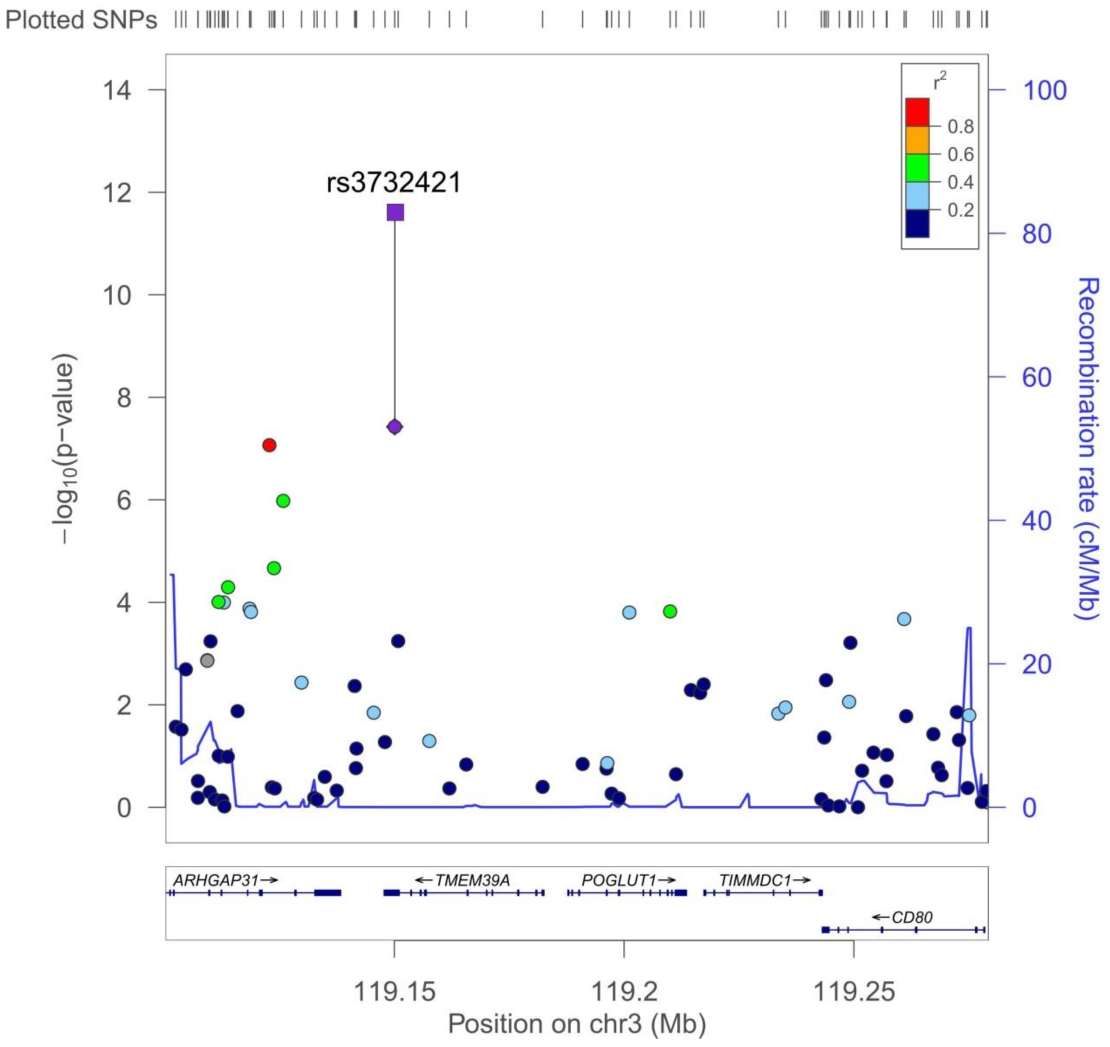
Plotted SNPs
rs3732421
r2
0.8
0.6
0.4
0.2
14
12
10
8
6
4
2
0
−log10(p−value)
100
80
60
40
20
0
Recombination rate (cM/Mb)
ARHGAP31→
←TMEM39A
POGLUT1→
TIMMDC1→
←CD80
119.15
119.2
119.25
Position on chr3 (Mb)

e. STAT1-STAT4 locus

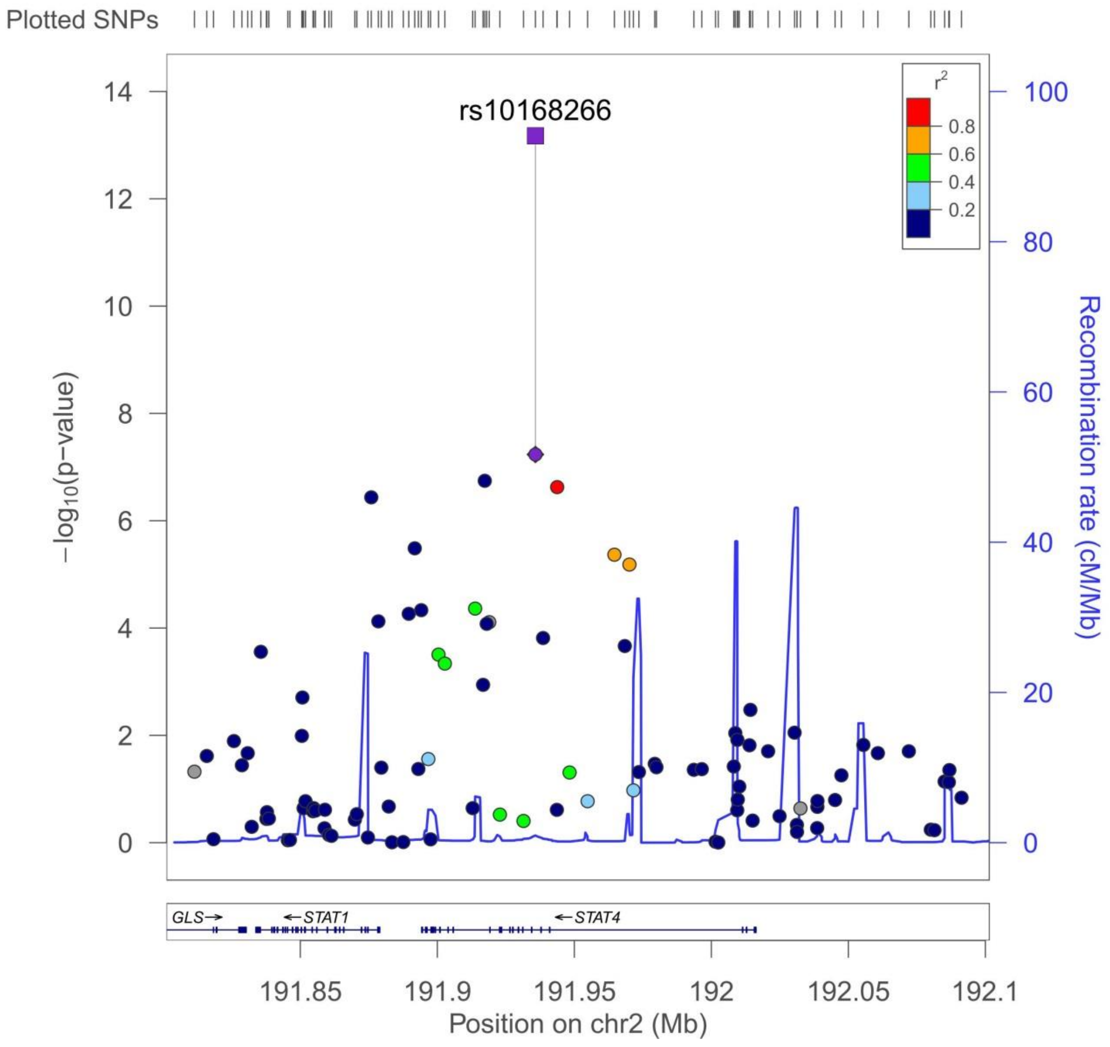

f. IL12A locus

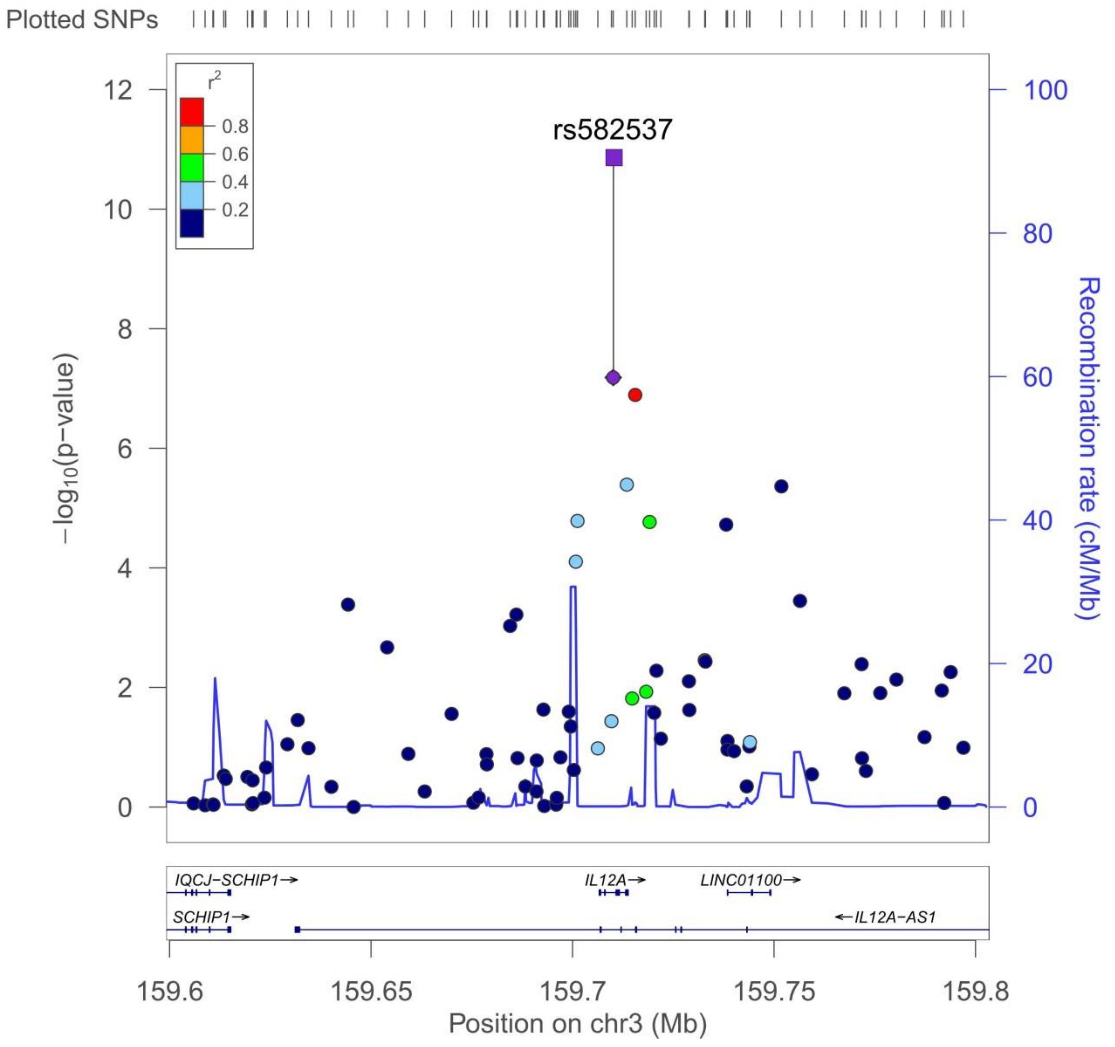

g. NF-κB locus

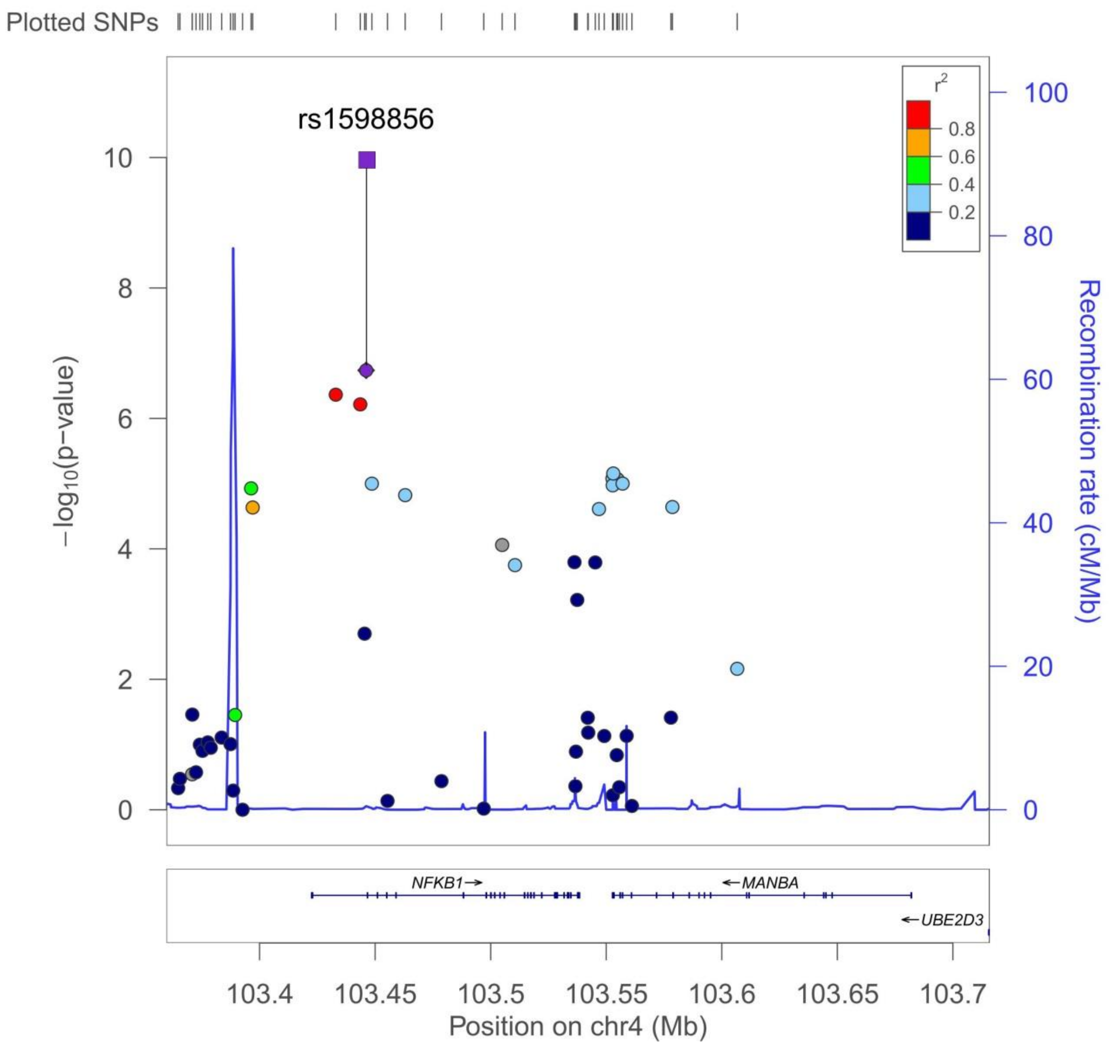

Plotted SNPs
rs1598856
r²
0.8
0.6
0.4
0.2
−log10(p−value)
10
8
6
4
2
0
100
80
60
40
20
0
Recombination rate (cM/Mb)
NFKB1→
←MANBA
←UBE2D3
103.4
103.45
103.5
103.55
103.6
103.65
103.7
Position on chr4 (Mb)

h. PDGFB-RPL3-SYNGR1 locus

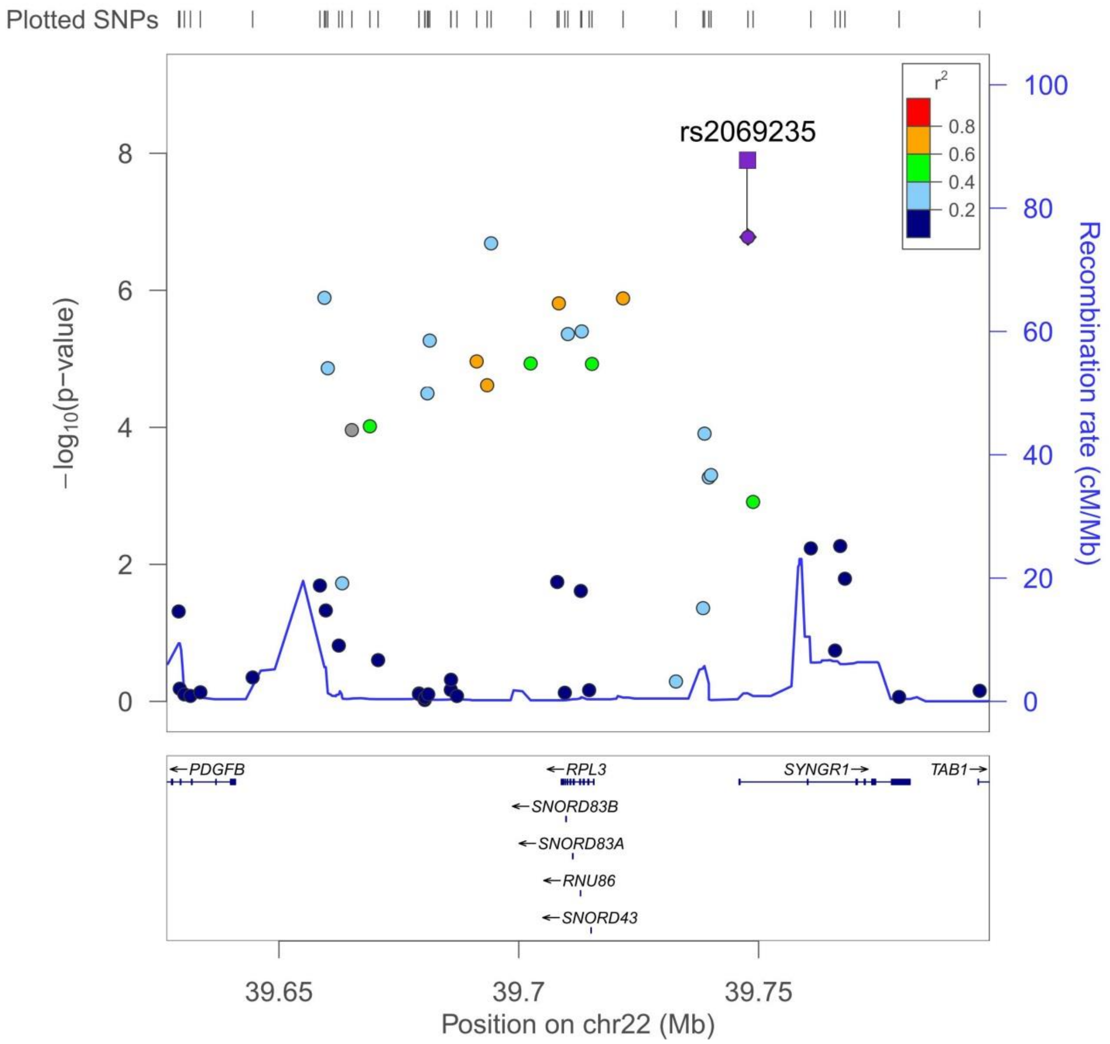

Supplementary Tables

Supplementary Table 1: Sample summary information for the GWAS and replication studies.

Study Stage	Total Samples	Disease	Female/Male	Average Age (±SD)	Genotyping Method
Discovery	1122	PBC	976/146	54.7 (22-85)	Illumina HumanOmniZhongHua-8 Beadchip (v1.1)
Discovery	4036	Control	1647/2389	34.8 (15-96)	Illumina HumanOmniZhongHua-8 Beadchip (v1.1)
Replication	907	PBC	763/144	56.1 (19-88)	Sequenom iPLEX
Replication	2127	Control	1636/491	50.7 (5-81)	Sequenom iPLEX

Supplementary Table 2: Demographics and clinical Features of PBC cases used in discovery and replication analysis

	GWAS	Replication
Number of PBC		
Male	146	144
Female	976	763
Age (years)		
range	17-85	19-88
median	55	51
mean (±SD)	54.7 (22-85)	56.1 (19-88)
AMA positivity (%)	96.47	96.17
gp210 positivity (%)	38.66	33.98
sp100 positivity (%)	23.84	22.44

Supplementary Table 3: SNP Filtering for Statistical Analysis

QC Parameter	Threshold	Remaining SNP	Excluded SNP
Mitochondrial SNP		894371	146
Sex Chromosome SNP		868834	25533
SNP call rate	98%	864671	4163
Minor allele frequency (MAF)	< 0.01	798519	66152
Hardy-Weinberg P value (HWP)	$P < 1.0 \times 10^{-4}$	776570	21949
SNP clustering	manual check	**776516**	54

Supplementary Table 4: SNPs from the HLA region reaching genome-wide significance in the discovery stage

CHR	SNP	BP*	Locus	Allele 1	MAF_case	MAF_control	Allele 2	P value	OR
6	rs9467715	26341301	*BTN3A2*	G	0.076	0.046	A	3.24E-08	1.69 (1.40 - 2.04)
6	rs9393736	26583676	*ABT1*	A	0.053	0.029	C	2.52E-08	1.88 (1.50 - 2.35)
6	rs12191629	26588461	*ABT1*	A	0.053	0.029	G	2.05E-08	1.88 (1.51 - 2.36)
6	rs12205310	26605864	*ABT1*	A	0.072	0.042	C	1.59E-09	1.80 (1.48 - 2.19)
6	rs10946842	26629233	*ZNF322*	A	0.072	0.042	C	3.01E-09	1.79 (1.47 - 2.17)
6	rs2853958	31233074	*HLA-C*	A	0.113	0.076	C	3.27E-08	1.55 (1.32 - 1.81)
6	rs3819299	31322367	*HLA-B*	C	0.111	0.074	A	2.27E-08	1.56 (1.33 - 1.82)
6	rs2523643	31342660	*MICA*	A	0.375	0.311	G	7.97E-09	1.33 (1.21 - 1.47)
6	rs2507987	31343033	*MICA*	A	0.395	0.332	T	3.72E-08	1.31 (1.19 - 1.44)
6	rs2507985	31343100	*MICA*	A	0.395	0.332	C	3.61E-08	1.31 (1.19 - 1.44)
6	rs5025316	31343574	*MICA*	G	0.395	0.332	A	3.46E-08	1.31 (1.19 - 1.44)
6	rs5025315	31343604	*MICA*	A	0.395	0.331	G	2.08E-08	1.32 (1.20 - 1.45)
6	rs3957110	31344348	*MICA*	A	0.395	0.332	G	2.70E-08	1.31 (1.19 - 1.45)
6	rs3132454	31489644	*MCCD1*	G	0.428	0.363	A	1.82E-08	1.31 (1.19 - 1.44)
6	rs3132451	31582025	*AIF1*	C	0.140	0.097	G	5.11E-09	1.52 (1.32 - 1.74)
6	rs3130070	31591808	*PRRC2A*	G	0.141	0.097	A	2.75E-09	1.52 (1.33 - 1.75)
6	rs3130622	31592524	*PRRC2A*	C	0.141	0.098	G	4.38E-09	1.52 (1.32 - 1.74)
6	rs3130623	31597700	*PRRC2A*	A	0.141	0.098	G	6.85E-09	1.51 (1.31 - 1.73)
6	rs3130626	31598489	*PRRC2A*	G	0.141	0.097	A	4.10E-09	1.52 (1.32 - 1.75)
6	rs2736157	31600820	*PRRC2A*	G	0.141	0.098	A	4.54E-09	1.52 (1.32 - 1.74)
6	rs3130627	31600851	*PRRC2A*	A	0.141	0.097	C	3.31E-09	1.52 (1.32 - 1.75)
6	rs3115663	31601843	*PRRC2A*	G	0.143	0.097	A	5.15E-10	1.55 (1.35 - 1.79)
6	rs9267525	31607942	*BAG6*	G	0.138	0.092	A	2.37E-10	1.58 (1.37 - 1.82)

6	rs3130628	31609272	*BAG6*	G	0.141	0.097	A	1.93E-09	1.53 (1.33 - 1.76)
6	rs3117583	31619576	*BAG6*	G	0.141	0.097	A	2.26E-09	1.53 (1.33 - 1.76)
6	rs3130618	31632134	*GPANK1*	A	0.142	0.095	C	3.25E-10	1.56 (1.36 - 1.80)
6	rs9267529	31634570	*GPANK1*	C	0.141	0.097	A	1.79E-09	1.53 (1.33 - 1.76)
6	rs2142234	31639129	*LY6G5B*	A	0.142	0.097	G	9.80E-10	1.54 (1.34 - 1.77)
6	rs9267532	31639979	*LY6G5B*	A	0.138	0.092	G	1.97E-10	1.58 (1.37 - 1.82)
6	rs9267533	31645922	*LY6G5C*	A	0.141	0.097	G	2.10E-09	1.53 (1.33 - 1.76)
6	rs9267534	31646766	*LY6G5C*	G	0.141	0.097	A	2.87E-09	1.52 (1.33 - 1.75)
6	rs9267535	31649149	*LY6G5C*	C	0.142	0.098	G	1.68E-09	1.53 (1.33 - 1.76)
6	rs9267536	31651194	*ABHD16A*	C	0.133	0.085	A	9.25E-12	1.65 (1.43 - 1.90)
6	rs405722	31685945	*LY6G6D*	A	0.141	0.097	C	1.88E-09	1.53 (1.33 - 1.76)
6	rs376510	31688200	*LY6G6C*	A	0.141	0.093	G	6.31E-11	1.59 (1.38 - 1.83)
6	rs400547	31702710	*CLIC1*	A	0.138	0.091	G	9.85E-11	1.59 (1.38 - 1.84)
6	rs707915	31710968	*MSH5*	A	0.141	0.094	T	1.02E-10	1.58 (1.38 - 1.82)
6	rs9267522	31711749	*MSH5*	G	0.141	0.097	A	3.00E-09	1.52 (1.32 - 1.75)
6	rs1150793	31717696	*MSH5*	G	0.140	0.093	A	1.47E-10	1.58 (1.37 - 1.82)
6	rs707934	31735631	*MSH5*	A	0.140	0.094	G	1.29E-10	1.58 (1.37 - 1.82)
6	rs10573	31763417	*VARS*	A	0.141	0.097	G	2.09E-09	1.53 (1.33 - 1.76)
6	rs16867582	31782617	*HSPA1L*	A	0.079	0.047	C	1.86E-09	1.75 (1.45 - 2.10)
6	rs12193582	31820755	*NEU1*	A	0.080	0.047	G	1.20E-09	1.76 (1.46 - 2.11)
6	rs11966200	31837066	*SLC44A4*	A	0.082	0.048	G	9.41E-10	1.75 (1.46 - 2.11)
6	rs3998219	31862388	*EHMT2*	A	0.069	0.040	G	1.08E-08	1.77 (1.45 - 2.15)
6	rs9267673	31883679	*C2*	A	0.158	0.112	G	3.02E-09	1.49 (1.31 - 1.70)
6	rs9267677	31892641	*C2*	G	0.154	0.108	A	1.20E-09	1.51 (1.32 - 1.73)
6	rs13209014	32127276	*PPT2*	A	0.082	0.048	G	1.04E-09	1.75 (1.46 - 2.10)
6	rs176095	32158319	*GPSM3*	G	0.296	0.227	A	1.63E-11	1.43 (1.29 - 1.59)
6	rs8192588	32188678	*NOTCH4*	A	0.082	0.049	G	2.03E-09	1.73 (1.45 - 2.08)

6	rs499606	32194308	*NOTCH4*	A	0.230	0.177	G	1.95E-08	1.38 (1.24 - 1.55)
6	rs117263199	32196066	*NOTCH4*	A	0.115	0.076	G	4.68E-09	1.58 (1.35 - 1.84)
6	rs17606000	32386204	*HLA-DRA*	A	0.036	0.073	T	2.63E-10	0.47 (0.37 - 0.60)
6	rs3135364	32389545	*HLA-DRA*	G	0.081	0.125	A	1.82E-08	0.62 (0.53 - 0.74)
6	rs3135358	32390748	*HLA-DRA*	A	0.067	0.120	G	6.75E-13	0.52 (0.44 - 0.63)
6	rs6929953	32391988	*HLA-DRA*	A	0.126	0.083	G	4.49E-10	1.59 (1.38 - 1.85)
6	rs6936170	32392821	*HLA-DRA*	A	0.126	0.084	G	7.97E-10	1.58 (1.37 - 1.83)
6	rs3135351	32392945	*HLA-DRA*	A	0.067	0.120	C	6.45E-13	0.52 (0.44 - 0.63)
6	rs6457579	32393093	*HLA-DRA*	G	0.126	0.084	A	9.89E-10	1.58 (1.36 - 1.83)
6	rs6457580	32393141	*HLA-DRA*	A	0.126	0.084	C	9.66E-10	1.58 (1.36 - 1.83)
6	rs7771308	32393259	*HLA-DRA*	A	0.126	0.084	G	8.69E-10	1.58 (1.36 - 1.83)
6	rs3135342	32396615	*HLA-DRA*	A	0.083	0.139	C	2.88E-12	0.56 (0.48 - 0.66)
6	rs3129851	32397712	*HLA-DRA*	C	0.094	0.165	A	1.25E-16	0.53 (0.45 - 0.62)
6	rs9268589	32398202	*HLA-DRA*	G	0.329	0.402	A	2.51E-10	0.73 (0.66 - 0.80)
6	rs7774047	32398563	*HLA-DRA*	C	0.328	0.402	A	2.62E-10	0.73 (0.66 - 0.80)
6	rs3129853	32398648	*HLA-DRA*	A	0.095	0.166	G	6.35E-17	0.53 (0.45 - 0.61)
6	rs4988822	32398975	*HLA-DRA*	A	0.095	0.166	G	9.19E-17	0.53 (0.45 - 0.61)
6	rs4988821	32399022	*HLA-DRA*	G	0.094	0.166	A	5.63E-17	0.52 (0.45 - 0.61)
6	rs4348358	32399092	*HLA-DRA*	G	0.328	0.402	A	2.38E-10	0.73 (0.66 - 0.80)
6	rs3135339	32399261	*HLA-DRA*	C	0.095	0.166	G	6.15E-17	0.53 (0.45 - 0.61)
6	rs9268605	32399833	*HLA-DRA*	G	0.329	0.402	A	2.93E-10	0.73 (0.66 - 0.80)
6	rs9268606	32400070	*HLA-DRA*	G	0.328	0.403	A	1.65E-10	0.73 (0.66 - 0.80)
6	rs3129858	32400520	*HLA-DRA*	A	0.097	0.167	G	5.39E-16	0.54 (0.46 - 0.63)
6	rs9268607	32400538	*HLA-DRA*	A	0.328	0.402	G	2.25E-10	0.73 (0.66 - 0.80)
6	rs9268608	32400865	*HLA-DRA*	G	0.328	0.403	A	1.78E-10	0.73 (0.66 - 0.80)
6	rs9268609	32401261	*HLA-DRA*	G	0.328	0.403	A	1.63E-10	0.73 (0.66 - 0.80)
6	rs7753264	32402440	*HLA-DRA*	A	0.328	0.402	G	2.11E-10	0.73 (0.66 - 0.80)

6	rs7773756	32402464	*HLA-DRA*	A	0.328	0.402	G	2.87E-10	0.73 (0.66 - 0.80)
6	rs9268613	32402692	*HLA-DRA*	G	0.329	0.402	A	3.66E-10	0.73 (0.66 - 0.81)
6	rs9268615	32402889	*HLA-DRA*	G	0.328	0.402	A	1.55E-10	0.73 (0.66 - 0.80)
6	rs3135398	32403934	*HLA-DRA*	A	0.095	0.166	G	8.81E-17	0.53 (0.45 - 0.61)
6	rs3135396	32404213	*HLA-DRA*	A	0.094	0.166	G	5.99E-17	0.53 (0.45 - 0.61)
6	rs2395174	32404878	*HLA-DRA*	C	0.094	0.165	A	1.19E-16	0.53 (0.45 - 0.62)
6	rs2395176	32405062	*HLA-DRA*	A	0.094	0.166	C	5.82E-17	0.52 (0.45 - 0.61)
6	rs2395177	32405076	*HLA-DRA*	C	0.095	0.166	G	9.12E-17	0.53 (0.45 - 0.61)
6	rs9268632	32406412	*HLA-DRA*	C	0.094	0.166	G	5.61E-17	0.52 (0.45 - 0.61)
6	rs9268634	32406530	*HLA-DRA*	G	0.327	0.402	A	1.33E-10	0.72 (0.66 - 0.80)
6	rs9268641	32406887	*HLA-DRA*	A	0.094	0.166	G	6.22E-17	0.53 (0.45 - 0.61)
6	rs3129872	32407153	*HLA-DRA*	A	0.094	0.166	T	6.07E-17	0.53 (0.45 - 0.61)
6	rs16822586	32407773	*HLA-DRA*	C	0.127	0.084	G	4.65E-10	1.59 (1.37 - 1.84)
6	rs9268644	32408044	*HLA-DRA*	A	0.098	0.177	C	2.41E-19	0.51 (0.44 - 0.59)
6	rs9268645	32408527	*HLA-DRA*	C	0.328	0.401	G	2.80E-10	0.73 (0.66 - 0.80)
6	rs3135393	32408842	*HLA-DRA*	G	0.074	0.136	A	1.52E-15	0.50 (0.43 - 0.60)
6	rs9268840	32428804	*HLA-DRA*	A	0.149	0.210	G	1.34E-10	0.66 (0.58 - 0.75)
6	rs9268854	32429672	*HLA-DRA*	G	0.148	0.210	A	7.14E-11	0.65 (0.58 - 0.74)
6	rs9268857	32429739	*HLA-DRA*	G	0.149	0.212	A	2.50E-11	0.65 (0.57 - 0.74)
6	rs9268859	32429804	*HLA-DRA*	C	0.148	0.210	A	5.58E-11	0.65 (0.57 - 0.74)
6	rs9268862	32430167	*HLA-DRA*	C	0.149	0.203	A	7.56E-09	0.69 (0.60 - 0.78)
6	rs7766854	32430752	*HLA-DRA*	A	0.149	0.210	G	1.15E-10	0.66 (0.58 - 0.75)
6	rs7746751	32430867	*HLA-DRA*	A	0.148	0.199	G	4.68E-08	0.70 (0.62 - 0.80)
6	rs7767493	32431129	*HLA-DRA*	A	0.151	0.215	G	1.47E-11	0.65 (0.57 - 0.73)
6	rs9268878	32431292	*HLA-DRA*	T	0.149	0.210	A	8.86E-11	0.66 (0.58 - 0.75)
6	rs9268879	32431306	*HLA-DRA*	C	0.148	0.210	A	1.01E-10	0.66 (0.58 - 0.75)
6	rs9268885	32431705	*HLA-DRA*	A	0.148	0.209	G	1.18E-10	0.66 (0.58 - 0.75)

6	rs9268886	32431828	*HLA-DRA*	A	0.148	0.210	G	1.09E-10	0.66 (0.58 - 0.75)
6	rs9268887	32431833	*HLA-DRA*	C	0.148	0.207	G	4.69E-10	0.67 (0.59 - 0.76)
6	rs9268907	32432233	*HLA-DRA*	G	0.148	0.210	A	1.01E-10	0.66 (0.58 - 0.75)
6	rs9268924	32432858	*HLA-DRA*	A	0.149	0.209	G	2.07E-10	0.66 (0.58 - 0.75)
6	rs2395188	32433256	*HLA-DRA*	G	0.149	0.210	A	1.15E-10	0.66 (0.58 - 0.75)
6	rs9268973	32434778	*HLA-DRA*	G	0.148	0.209	A	1.18E-10	0.66 (0.58 - 0.75)
6	rs9268974	32434830	*HLA-DRA*	A	0.149	0.209	G	1.62E-10	0.66 (0.58 - 0.75)
6	rs9268977	32434939	*HLA-DRA*	G	0.148	0.209	A	1.22E-10	0.66 (0.58 - 0.75)
6	rs9268980	32435123	*HLA-DRA*	G	0.149	0.210	A	1.05E-10	0.66 (0.58 - 0.75)
6	rs9269043	32438598	*HLA-DRA*	G	0.148	0.209	A	1.04E-10	0.66 (0.58 - 0.75)
6	rs7452863	32439068	*HLA-DRA*	C	0.147	0.208	G	1.18E-10	0.66 (0.58 - 0.75)
6	rs9286789	32439696	*HLA-DRA*	C	0.071	0.116	A	1.37E-09	0.58 (0.49 - 0.70)
6	rs9461755	32441046	*HLA-DRA*	A	0.071	0.119	G	1.16E-10	0.57 (0.48 - 0.67)
6	rs6457597	32444455	*HLA-DRA*	G	0.148	0.210	A	7.78E-11	0.65 (0.58 - 0.74)
6	rs7748435	32448652	*HLA-DRA*	G	0.149	0.208	A	4.24E-10	0.67 (0.59 - 0.76)
6	rs7748494	32448808	*HLA-DRA*	G	0.070	0.118	A	1.80E-10	0.57 (0.48 - 0.68)
6	rs2395200	32451888	*HLA-DRA*	G	0.142	0.212	A	1.41E-13	0.61 (0.54 - 0.70)
6	rs35759989	32581096	*HLA-DQA1*	G	0.157	0.113	A	3.94E-08	1.45 (1.27 - 1.66)
6	rs11751024	32586236	*HLA-DQA1*	A	0.389	0.325	C	1.64E-08	1.32 (1.20 - 1.46)
6	rs9271573	32590501	*HLA-DQA1*	A	0.328	0.409	C	3.10E-12	0.70 (0.64 - 0.78)
6	rs9271640	32592200	*HLA-DQA1*	A	0.196	0.252	G	4.42E-08	0.72 (0.65 - 0.81)
6	rs3129768	32595083	*HLA-DQA1*	C	0.196	0.253	A	2.93E-08	0.72 (0.64 - 0.81)
6	rs9272219	32602269	*HLA-DQA1*	A	0.197	0.258	C	2.56E-09	0.71 (0.63 - 0.79)
6	rs3104369	32602482	*HLA-DQA1*	A	0.204	0.267	G	1.50E-09	0.70 (0.63 - 0.79)
6	rs9272783	32610378	*HLA-DQA1*	A	0.197	0.258	G	2.74E-09	0.71 (0.63 - 0.79)
6	rs9273012	32611641	*HLA-DQA1*	G	0.197	0.258	A	2.94E-09	0.71 (0.63 - 0.79)
6	rs17212090	32650809	*HLA-DQB1*	A	0.367	0.447	G	1.44E-11	0.72 (0.65 - 0.79)

6	rs9275144	32651218	*HLA-DQB1*	T	0.376	0.312	A	9.15E-09	1.33 (1.21 - 1.47)
6	rs3021061	32651839	*HLA-DQB1*	G	0.376	0.313	C	1.27E-08	1.33 (1.20 - 1.46)
6	rs17206012	32652077	*HLA-DQB1*	C	0.371	0.449	A	5.21E-11	0.73 (0.66 - 0.80)
6	rs4947342	32653070	*HLA-DQB1*	A	0.382	0.319	G	1.29E-08	1.33 (1.20 - 1.46)
6	rs2856692	32653385	*HLA-DQB1*	C	0.382	0.320	A	4.90E-08	1.31 (1.19 - 1.44)
6	rs2856688	32654640	*HLA-DQB1*	A	0.382	0.318	C	1.47E-08	1.32 (1.20 - 1.46)
6	rs28747027	32654977	*HLA-DQB1*	G	0.361	0.439	C	5.34E-11	0.72 (0.66 - 0.80)
6	rs113201741	32657227	*HLA-DQB1*	G	0.047	0.085	A	2.47E-09	0.53 (0.43 - 0.66)
6	rs3129713	32657255	*HLA-DQB1*	A	0.319	0.243	G	3.61E-13	1.46 (1.32 - 1.62)
6	rs7774434	32657578	*HLA-DQB1*	G	0.462	0.390	A	5.91E-10	1.35 (1.23 - 1.48)
6	rs7775228	32658079	*HLA-DQB1*	G	0.321	0.247	A	1.92E-12	1.44 (1.30 - 1.60)
6	rs9469219	32658211	*HLA-DQB1*	A	0.558	0.482	G	2.50E-10	1.35 (1.23 - 1.49)
6	rs3129718	32659838	*HLA-DQB1*	A	0.050	0.093	G	9.26E-11	0.51 (0.42 - 0.63)
6	rs9275224	32659878	*HLA-DQB1*	A	0.469	0.377	G	2.11E-15	1.46 (1.33 - 1.61)
6	rs9275245	32660943	*HLA-DQB1*	A	0.470	0.377	G	2.30E-15	1.46 (1.33 - 1.61)
6	rs9275273	32662559	*HLA-DQB1*	C	0.500	0.417	A	1.42E-12	1.40 (1.28 - 1.54)
6	rs2647017	32663772	*HLA-DQB1*	C	0.248	0.180	A	6.66E-13	1.50 (1.34 - 1.68)
6	rs2647015	32664093	*HLA-DQB1*	C	0.247	0.178	A	1.97E-13	1.52 (1.36 - 1.70)
6	rs9275382	32668831	*HLA-DQB1*	G	0.104	0.151	A	1.61E-08	0.65 (0.56 - 0.76)
6	rs2858308	32670000	*HLA-DQB1*	A	0.248	0.178	C	2.16E-13	1.52 (1.36 - 1.69)
6	rs9501224	32792910	*TAP2*	A	0.492	0.427	G	4.47E-08	1.30 (1.18 - 1.43)
6	rs12197929	32889593	*HLA-DMB*	G	0.077	0.043	A	5.94E-11	1.86 (1.54 - 2.25)
6	rs12206426	32902609	*HLA-DMB*	C	0.072	0.040	A	2.34E-10	1.86 (1.53 - 2.26)
6	rs171329	32904661	*HLA-DMB*	A	0.317	0.386	G	1.98E-09	0.74 (0.67 - 0.82)
6	rs6934645	32906228	*HLA-DMB*	A	0.071	0.039	C	3.47E-10	1.86 (1.53 - 2.26)
6	rs12206377	32909335	*HLA-DMB*	G	0.071	0.039	A	3.05E-10	1.86 (1.53 - 2.27)
6	rs3918154	32943752	*BRD2*	G	0.071	0.039	A	2.95E-10	1.86 (1.53 - 2.27)

6	rs10456417	32988651	*HLA-DOA*	G	0.074	0.038	A	1.02E-12	2.01 (1.65 - 2.44)
6	rs6914052	33003119	*HLA-DOA*	A	0.074	0.038	G	7.15E-13	2.02 (1.66 - 2.45)
6	rs430620	33017486	*HLA-DPA1*	A	0.097	0.061	G	2.62E-09	1.65 (1.40 - 1.95)
6	rs9380335	33025174	*HLA-DPA1*	C	0.283	0.344	G	4.74E-08	0.75 (0.68 - 0.83)
6	rs2395309	33026246	*HLA-DPA1*	A	0.282	0.344	G	3.33E-08	0.75 (0.68 - 0.83)
6	rs34937821	33026553	*HLA-DPA1*	G	0.283	0.351	A	1.73E-09	0.73 (0.66 - 0.81)
6	rs7751953	33033351	*HLA-DPA1*	C	0.283	0.346	A	2.88E-08	0.75 (0.68 - 0.83)
6	rs3180553	33036210	*HLA-DPA1*	C	0.283	0.346	A	2.78E-08	0.75 (0.68 - 0.83)
6	rs1042190	33036999	*HLA-DPA1*	A	0.307	0.375	G	2.11E-09	0.74 (0.67 - 0.81)
6	rs1042174	33037626	*HLA-DPA1*	C	0.306	0.371	G	1.73E-08	0.75 (0.68 - 0.83)
6	rs2301224	33038369	*HLA-DPA1*	C	0.307	0.375	A	2.33E-09	0.74 (0.67 - 0.81)
6	rs1431399	33041034	*HLA-DPA1*	A	0.307	0.375	G	2.56E-09	0.74 (0.67 - 0.82)
6	rs1431401	33041186	*HLA-DPA1*	G	0.307	0.375	A	2.96E-09	0.74 (0.67 - 0.82)
6	rs2071351	33043930	*HLA-DPB1*	A	0.283	0.344	G	4.21E-08	0.75 (0.68 - 0.83)
6	rs2567281	33047898	*HLA-DPB1*	A	0.082	0.048	C	7.82E-10	1.76 (1.47 - 2.11)
6	rs1042169	33048686	*HLA-DPB1*	G	0.320	0.394	A	2.00E-10	0.73 (0.66 - 0.80)
6	rs9277356	33048694	*HLA-DPB1*	A	0.320	0.403	G	9.17E-13	0.70 (0.63 - 0.77)
6	rs928976	33049211	*HLA-DPB1*	G	0.333	0.404	A	1.21E-09	0.74 (0.67 - 0.81)
6	rs9501251	33049663	*HLA-DPB1*	G	0.082	0.044	A	8.18E-13	1.94 (1.61 - 2.33)
6	rs9500928	33049694	*HLA-DPB1*	A	0.082	0.044	G	8.18E-13	1.94 (1.61 - 2.33)
6	rs16868943	33147727	*COL11A2*	A	0.066	0.030	G	6.07E-15	2.26 (1.83 - 2.78)

*Sequence position was annotated based on GRCh37/hg19 Assembly. Chr., chromosome; MAF, minor allele frequency.
P value is based on additive model; OR, odds ratio, is calculated for minor allele

Supplementary Table 5: SNPs from non-HLA genome-wide significant genomic regions with P value < 1×10^{-4} in the discovery stage

CHR	SNP	Position*	Locus	Allele 1	MAF (case)	MAF (control)	Allele 2	P value#	OR
1p13.1	rs17035862	117032885	*CD58*	G	0.517	0.462	C	3.03E-06	1.25 (1.14 - 1.37)
1p13.1	rs10754444	117049676	*CD58*	A	0.482	0.427	G	2.69E-06	1.25 (1.14 - 1.38)
1p13.1	rs10924106	117053745	*CD58*	A	0.509	0.450	G	4.75E-07	1.27 (1.16 - 1.40)
1p13.1	rs10924108	117062474	*CD58*	A	0.509	0.449	G	4.58E-07	1.27 (1.16 - 1.40)
1p13.1	rs1414275	117066907	*CD58*	A	0.509	0.449	G	8.38E-07	1.27 (1.16 - 1.40)
1p13.1	rs6677309	117080166	*CD58*	A	0.480	0.423	C	1.44E-06	1.26 (1.15 - 1.38)
1p13.1	rs758518	117081810	*CD58*	A	0.507	0.449	G	1.10E-06	1.26 (1.15 - 1.39)
1p13.1	rs12044852	117087779	*CD58*	C	0.510	0.452	A	1.20E-06	1.26 (1.15 - 1.38)
1p13.1	rs2300747	117104215	*CD58*	A	0.481	0.416	G	4.54E-08	1.30 (1.18 - 1.43)
2q32.3	rs13029532	191875901	*STAT1, STAT4*	C	0.052	0.084	A	3.66E-07	0.59 (0.49 - 0.73)
2q32.3	rs41430444	191878487	*STAT1, STAT4*	G	0.102	0.133	A	7.50E-05	0.74 (0.63 - 0.86)
2q32.3	rs6740131	191889582	*STAT1, STAT4*	G	0.186	0.226	A	5.42E-05	0.78 (0.70 - 0.88)
2q32.3	rs3024918	191891763	*STAT1, STAT4*	G	0.120	0.160	A	3.26E-06	0.72 (0.62 - 0.83)
2q32.3	rs3024908	191894141	*STAT1, STAT4*	G	0.110	0.144	A	4.64E-05	0.74 (0.64 - 0.85)
2q32.3	rs16833215	191913799	*STAT1, STAT4*	G	0.504	0.455	A	4.33E-05	1.22 (1.11 - 1.34)
2q32.3	rs4853540	191917317	*STAT1, STAT4*	A	0.075	0.114	C	1.80E-07	0.64 (0.54 - 0.75)
2q32.3	rs11686127	191918031	*STAT1, STAT4*	A	0.055	0.080	G	8.32E-05	0.67 (0.55 - 0.82)
2q32.3	rs199509833	191918961	*STAT1, STAT4*	G	0.055	0.080	A	7.76E-05	0.67 (0.55 - 0.82)
2q32.3	rs10168266	191935804	*STAT1, STAT4*	A	0.397	0.336	G	5.82E-08	1.31 (1.19 - 1.44)
2q32.3	rs11889341	191943742	*STAT1, STAT4*	A	0.395	0.337	G	2.36E-07	1.29 (1.17 - 1.42)
2q32.3	rs7574865	191964633	*STAT1, STAT4*	A	0.389	0.337	C	4.29E-06	1.25 (1.14 - 1.38)
2q32.3	rs7582694	191970120	*STAT1, STAT4*	C	0.390	0.339	G	6.55E-06	1.25 (1.13 - 1.38)

2q32.3	rs1879877	204570000	*CD28, CTLA4*	C	0.331	0.384	A	5.12E-06	0.80 (0.72 - 0.88)
2q32.3	rs1181388	204575951	*CD28, CTLA4*	G	0.404	0.459	A	4.22E-06	0.80 (0.73 - 0.88)
2q32.3	rs10932020	204633156	*CD28, CTLA4*	A	0.408	0.483	G	4.30E-10	0.74 (0.67 - 0.81)
2q32.3	rs231390	204634767	*CD28, CTLA4*	A	0.312	0.375	G	3.98E-08	0.76 (0.68 - 0.84)
2q32.3	rs4673268	204639764	*CD28, CTLA4*	A	0.470	0.406	C	5.30E-08	1.30 (1.18 - 1.43)
2q32.3	rs4675369	204643194	*CD28, CTLA4*	G	0.526	0.449	A	6.56E-11	1.37 (1.24 - 1.50)
2q32.3	rs7599230	204648661	*CD28, CTLA4*	G	0.471	0.408	A	9.08E-08	1.29 (1.18 - 1.42)
2q32.3	rs16840128	204661621	*CD28, CTLA4*	G	0.472	0.413	A	4.02E-07	1.28 (1.16 - 1.40)
2q32.3	rs7591187	204676238	*CD28, CTLA4*	A	0.397	0.469	G	1.42E-09	0.75 (0.68 - 0.82)
2q32.3	rs17594516	204678417	*CD28, CTLA4*	A	0.467	0.410	G	1.19E-06	1.26 (1.15 - 1.39)
2q32.3	rs1018361	204684835	*CD28, CTLA4*	A	0.393	0.452	G	7.27E-07	0.79 (0.71 - 0.86)
2q32.3	rs231747	204688432	*CD28, CTLA4*	A	0.462	0.410	C	1.25E-05	1.23 (1.12 - 1.36)
2q32.3	rs78707218	204710197	*CD28, CTLA4*	A	0.446	0.399	G	5.25E-05	1.22 (1.11 - 1.34)
2q32.3	rs11571291	204721132	*CD28, CTLA4*	G	0.157	0.194	A	7.01E-05	0.77 (0.68 - 0.88)
2q32.3	rs11571315	204730901	*CD28, CTLA4*	G	0.286	0.334	A	2.14E-05	0.80 (0.72 - 0.89)
2q32.3	rs733618	204730944	*CD28, CTLA4*	G	0.447	0.399	A	4.12E-05	1.22 (1.11 - 1.34)
2q32.3	rs231775	204732714	*CD28, CTLA4*	A	0.286	0.334	G	1.49E-05	0.80 (0.72 - 0.88)
3q13.33	rs9831023	119111762	*CD80*	G	0.380	0.426	A	9.85E-05	0.83 (0.75 - 0.91)
3q13.33	rs1000198	119113820	*CD80*	A	0.436	0.485	C	5.09E-05	0.82 (0.75 - 0.90)
3q13.33	rs12494314	119122820	*CD80*	G	0.284	0.344	A	8.59E-08	0.76 (0.68 - 0.84)
3q13.33	rs6773050	119123814	*CD80*	G	0.439	0.489	A	2.16E-05	0.82 (0.74 - 0.90)
3q13.33	rs11922594	119125822	*CD80*	A	0.401	0.459	G	1.05E-06	0.79 (0.72 - 0.87)
3q13.33	rs3732421	119150089	*CD80*	G	0.271	0.332	A	3.76E-08	0.75 (0.67 - 0.83)
3q13.33	rs3830649	119246386	*CD80*	A	0.269	0.330	G	5.06E-08	0.75 (0.68 - 0.83)
3q25.33	rs2561284	159700819	*IL12A*	A	0.091	0.121	C	7.88E-05	0.73 (0.62 - 0.85)
3q25.33	rs662959	159701231	*IL12A*	A	0.093	0.126	G	1.64E-05	0.71 (0.61 - 0.83)
3q25.33	rs582537	159710098	*IL12A*	C	0.234	0.291	A	6.55E-08	0.74 (0.66 - 0.83)

3q25.33	rs568408	159713467	*IL12A*	A	0.096	0.132	G	4.05E-06	0.70 (0.60 - 0.81)
3q25.33	rs668998	159715551	*IL12A*	A	0.233	0.289	G	1.28E-07	0.75 (0.67 - 0.83)
3q25.33	rs485497	159719132	*IL12A*	G	0.206	0.250	A	1.71E-05	0.78 (0.70 - 0.87)
3q25.33	rs17289236	159738134	*IL12A*	A	0.081	0.113	G	1.89E-05	0.70 (0.59 - 0.82)
3q25.33	rs6783320	159751822	*IL12A*	A	0.216	0.264	G	4.33E-06	0.77 (0.69 - 0.86)
4q24	rs2903281	103396333	*NF-κB*	A	0.343	0.295	G	1.18E-05	1.25 (1.13 - 1.38)
4q24	rs2085548	103397014	*NF-κB*	A	0.396	0.347	G	2.32E-05	1.23 (1.12 - 1.36)
4q24	rs17032705	103432974	*NF-κB*	A	0.464	0.405	G	4.32E-07	1.27 (1.16 - 1.40)
4q24	rs1599961	103443569	*NF-κB*	A	0.464	0.405	G	6.09E-07	1.27 (1.16 - 1.40)
4q24	rs1598856	103446115	*NF-κB*	G	0.536	0.474	A	1.83E-07	1.28 (1.17 - 1.41)
4q24	rs230535	103448582	*NF-κB*	A	0.452	0.400	C	1.00E-05	1.24 (1.13 - 1.36)
4q24	rs118882	103463007	*NF-κB*	A	0.451	0.400	G	1.50E-05	1.23 (1.12 - 1.35)
4q24	rs201335879	103504964	*NF-κB*	A	0.501	0.455	G	8.71E-05	1.21 (1.10 - 1.32)
4q24	rs12645469	103546855	*NF-κB*	A	0.311	0.266	G	2.45E-05	1.25 (1.13 - 1.38)
4q24	rs1054037	103552709	*NF-κB*	G	0.447	0.500	A	8.35E-06	0.81 (0.74 - 0.89)
4q24	rs4013	103552813	*NF-κB*	G	0.447	0.499	A	1.06E-05	0.81 (0.74 - 0.89)
4q24	rs1054029	103553053	*NF-κB*	G	0.447	0.501	A	6.98E-06	0.81 (0.73 - 0.89)
4q24	rs75707396	103554932	*NF-κB*	C	0.446	0.499	A	8.59E-06	0.81 (0.74 - 0.89)
4q24	rs2866413	103557077	*NF-κB*	G	0.447	0.500	A	9.97E-06	0.81 (0.74 - 0.89)
4q24	rs228614	103578637	*NF-κB*	G	0.520	0.469	A	2.28E-05	1.22 (1.12 - 1.34)
4q27	rs10857092	123389219	*IL21*	A	0.425	0.378	G	6.40E-05	1.21 (1.10 - 1.33)
4q27	rs17005934	123549699	*IL21*	G	0.408	0.352	A	1.42E-06	1.27 (1.15 - 1.39)
4q27	rs62324212	123560939	*IL21*	A	0.492	0.442	C	2.26E-05	1.22 (1.12 - 1.34)
4q27	rs28517551	123561459	*IL21*	A	0.417	0.365	G	5.20E-06	1.25 (1.13 - 1.37)
4q27	rs147078612	123579975	*IL21*	C	0.447	0.503	A	2.28E-06	0.80 (0.73 - 0.88)
4q27	rs925550	123588526	*IL21*	A	0.428	0.370	C	6.21E-07	1.27 (1.16 - 1.40)
4q27	rs60318098	123599898	*IL21*	G	0.424	0.368	A	1.30E-06	1.26 (1.15 - 1.39)

4q27	rs6836610	123601697	*IL21*	A	0.505	0.455	G	2.87E-05	1.22 (1.11 - 1.34)
4q27	rs1026157	123623893	*IL21*	C	0.363	0.314	A	1.07E-05	1.25 (1.13 - 1.38)
4q27	rs416556	123629427	*IL21*	C	0.411	0.363	A	3.96E-05	1.22 (1.11 - 1.34)
4q27	rs1512974	123631308	*IL21*	G	0.413	0.365	A	3.19E-05	1.22 (1.11 - 1.35)
4q27	rs309370	123664204	*IL21*	G	0.412	0.361	A	1.34E-05	1.24 (1.12 - 1.36)
4q27	rs2292493	123664445	*IL21*	G	0.413	0.364	A	1.88E-05	1.23 (1.12 - 1.36)
4q27	rs4833843	123665428	*IL21*	G	0.414	0.365	A	1.71E-05	1.23 (1.12 - 1.36)
4q27	rs4833844	123668544	*IL21*	G	0.414	0.365	A	2.08E-05	1.23 (1.12 - 1.35)
4q27	rs6811183	123671551	*IL21*	A	0.413	0.362	C	9.81E-06	1.24 (1.13 - 1.36)
9q32	rs7856856	117540910	*TNFSF8, TNFSF15*	G	0.344	0.278	A	1.27E-09	1.36 (1.23 - 1.50)
9q32	rs6478106	117545666	*TNFSF8, TNFSF15*	A	0.342	0.277	G	1.61E-09	1.36 (1.23 - 1.50)
9q32	rs6478108	117558703	*TNFSF8, TNFSF15*	G	0.447	0.508	A	3.39E-07	0.78 (0.71 - 0.86)
9q32	rs4263839	117566440	*TNFSF8, TNFSF15*	A	0.439	0.501	G	2.98E-07	0.78 (0.71 - 0.86)
9q32	rs7862325	117567137	*TNFSF8, TNFSF15*	G	0.046	0.072	A	1.00E-05	0.62 (0.50 - 0.77)
9q32	rs7862325	117567137	*TNFSF8, TNFSF15*	G	0.046	0.072	A	1.00E-05	0.62 (0.50 - 0.77)
9q32	rs6478109	117568766	*TNFSF8, TNFSF15*	A	0.442	0.504	G	2.32E-07	0.78 (0.71 - 0.86)
9q32	rs7848647	117569046	*TNFSF8, TNFSF15*	A	0.443	0.503	G	3.15E-07	0.78 (0.71 - 0.86)
9q32	rs7866342	117627569	*TNFSF8, TNFSF15*	C	0.389	0.451	A	1.43E-07	0.77 (0.70 - 0.85)
9q32	rs7048073	117629689	*TNFSF8, TNFSF15*	A	0.016	0.032	G	3.69E-05	0.48 (0.34 - 0.69)
9q32	rs4979467	117630043	*TNFSF8, TNFSF15*	A	0.399	0.322	G	8.28E-12	1.40 (1.27 - 1.54)
9q32	rs7874896	117636879	*TNFSF8, TNFSF15*	A	0.016	0.031	C	5.80E-05	0.49 (0.34 - 0.70)
9q32	rs7028891	117645015	*TNFSF8, TNFSF15*	G	0.359	0.284	A	1.04E-11	1.41 (1.28 - 1.55)
9q32	rs1407309	117651780	*TNFSF8, TNFSF15*	A	0.410	0.333	G	1.43E-11	1.39 (1.26 - 1.53)
9q32	rs911605	117654990	*TNFSF8, TNFSF15*	G	0.410	0.467	A	1.08E-06	0.79 (0.72 - 0.87)
9q32	rs1322067	117660933	*TNFSF8, TNFSF15*	G	0.412	0.335	A	2.06E-11	1.39 (1.26 - 1.53)
9q32	rs2295800	117664211	*TNFSF8, TNFSF15*	G	0.415	0.337	A	9.03E-12	1.40 (1.27 - 1.54)
9q32	rs3181372	117665435	*TNFSF8, TNFSF15*	G	0.482	0.411	A	1.71E-09	1.33 (1.21 - 1.47)

9q32	rs3181369	117665866	*TNFSF8, TNFSF15*	A	0.313	0.261	G	7.15E-07	1.29 (1.17 - 1.43)
9q32	rs3181195	117668142	*TNFSF8, TNFSF15*	A	0.312	0.260	G	8.11E-07	1.29 (1.17 - 1.43)
9q32	rs1555457	117674320	*TNFSF8, TNFSF15*	A	0.293	0.226	G	7.32E-11	1.42 (1.27 - 1.57)
9q32	rs201804223	117678054	*TNFSF8, TNFSF15*	C	0.478	0.407	A	1.83E-09	1.33 (1.21 - 1.47)
9q32	rs4979472	117685398	*TNFSF8, TNFSF15*	G	0.477	0.408	A	4.22E-09	1.32 (1.21 - 1.46)
9q32	rs726657	117696336	*TNFSF8, TNFSF15*	A	0.310	0.260	G	2.92E-06	1.28 (1.15 - 1.41)
11q23.3	rs573905	118572267	*DDX6, CXCR5*	A	0.364	0.319	G	5.07E-05	1.22 (1.11 - 1.35)
11q23.3	rs4639966	118573519	*DDX6, CXCR5*	G	0.362	0.316	A	3.02E-05	1.23 (1.12 - 1.36)
11q23.3	rs480958	118577990	*DDX6, CXCR5*	G	0.423	0.366	A	1.02E-06	1.27 (1.15 - 1.39)
11q23.3	rs2077579	118619047	*DDX6, CXCR5*	C	0.103	0.140	A	4.22E-06	0.70 (0.61 - 0.82)
11q23.3	rs149737425	118620067	*DDX6, CXCR5*	A	0.161	0.114	G	4.69E-09	1.48 (1.30 - 1.69)
11q23.3	rs636736	118705145	*DDX6, CXCR5*	G	0.172	0.127	A	2.68E-08	1.44 (1.26 - 1.63)
11q23.3	rs7105658	118712270	*DDX6, CXCR5*	A	0.181	0.134	G	1.70E-08	1.43 (1.26 - 1.62)
11q23.3	rs77871618	118733624	*DDX6, CXCR5*	A	0.225	0.158	G	9.12E-14	1.55 (1.38 - 1.74)
11q23.3	rs4938572	118740931	*DDX6, CXCR5*	G	0.057	0.088	A	1.95E-06	0.63 (0.51 - 0.76)
11q23.3	rs12365699	118743286	*DDX6, CXCR5*	A	0.029	0.051	G	1.32E-05	0.56 (0.43 - 0.73)
11q23.3	rs61150131	118752178	*DDX6, CXCR5*	G	0.214	0.166	A	1.77E-07	1.36 (1.21 - 1.53)
11q23.3	rs75646772	118753597	*DDX6, CXCR5*	A	0.162	0.130	G	8.31E-05	1.30 (1.14 - 1.48)
12p13.31	rs1800693	6440009	*TNFRSF1A*	G	0.144	0.113	A	6.42E-05	1.32 (1.15 - 1.51)
12p13.31	rs4149576	6449115	*TNFRSF1A*	A	0.148	0.113	G	1.11E-05	1.35 (1.18 - 1.55)
12p13.31	rs118191630	6454373	*TNFRSF1A*	C	0.051	0.032	A	2.32E-05	1.62 (1.29 - 2.03)
15q25.1	rs11857713	81591265	*IL16*	A	0.232	0.188	G	3.74E-06	1.30 (1.17 - 1.46)
15q25.1	rs4778636	81591639	*IL16*	A	0.232	0.188	G	2.93E-06	1.31 (1.17 - 1.46)
15q25.1	rs11073001	81592802	*IL16*	G	0.269	0.224	A	8.21E-06	1.28 (1.15 - 1.42)
15q25.1	rs11556218	81598269	*IL16*	C	0.233	0.189	A	3.03E-06	1.31 (1.17 - 1.46)
15q25.1	rs1803275	81598416	*IL16*	A	0.233	0.189	G	3.43E-06	1.31 (1.17 - 1.46)
15q25.1	rs11325	81601340	*IL16*	A	0.282	0.234	C	2.98E-06	1.29 (1.16 - 1.43)

15q25.1	rs4778640	81604131	*IL16*	G	0.253	0.209	A	9.42E-06	1.28 (1.15 - 1.43)
16p12.1	rs1859308	27397998	*IL4R, IL21R*	A	0.267	0.321	G	9.88E-07	0.77 (0.69 - 0.86)
16p12.1	rs201883233	27398068	*IL4R, IL21R*	C	0.381	0.443	A	1.24E-07	0.77 (0.70 - 0.85)
16p12.1	rs10852316	27398555	*IL4R, IL21R*	A	0.379	0.443	C	8.83E-08	0.77 (0.70 - 0.85)
16p12.1	rs2189521	27413566	*IL4R, IL21R*	G	0.242	0.306	A	5.40E-09	0.73 (0.65 - 0.81)
16p12.1	rs58579343	27421927	*IL4R, IL21R*	G	0.315	0.375	A	1.39E-07	0.77 (0.69 - 0.85)
16q21	rs2731783	58253460	*CSNK2A2, CCDC113*	A	0.389	0.337	G	3.99E-06	1.26 (1.14 - 1.38)
16q21	rs2550374	58254448	*CSNK2A2, CCDC113*	A	0.424	0.484	C	4.19E-07	0.78 (0.71 - 0.86)
16q21	rs11647151	58258237	*CSNK2A2, CCDC113*	A	0.389	0.337	C	5.35E-06	1.25 (1.14 - 1.38)
16q21	rs2731773	58259467	*CSNK2A2, CCDC113*	A	0.267	0.315	G	1.51E-05	0.79 (0.72 - 0.88)
16q21	rs1820235	58264412	*CSNK2A2, CCDC113*	A	0.421	0.475	G	4.33E-06	0.80 (0.73 - 0.88)
17q12	rs2643194	37853048	*ORMDL3, ZPBP2, GSDMB, IKZF3*	A	0.449	0.403	G	7.63E-05	1.21 (1.10 - 1.33)
17q12	rs907091	37921742	*ORMDL3, ZPBP2, GSDMB, IKZF3*	G	0.361	0.294	A	1.88E-09	1.35 (1.23 - 1.49)
17q12	rs10445308	37938047	*ORMDL3, ZPBP2, GSDMB, IKZF3*	A	0.359	0.293	G	1.97E-09	1.35 (1.23 - 1.49)
17q12	rs9303277	37976469	*ORMDL3, ZPBP2, GSDMB, IKZF3*	A	0.358	0.292	G	2.53E-09	1.35 (1.22 - 1.49)
17q12	rs9635726	38020141	*ORMDL3, ZPBP2, GSDMB, IKZF3*	G	0.382	0.309	A	6.42E-11	1.38 (1.25 - 1.52)
17q12	rs12150079	38025417	*ORMDL3, ZPBP2, GSDMB, IKZF3*	A	0.244	0.195	G	4.59E-07	1.33 (1.19 - 1.49)
17q12	rs11557467	38028634	*ORMDL3, ZPBP2, GSDMB, IKZF3*	A	0.328	0.261	C	2.56E-10	1.38 (1.25 - 1.53)
17q12	rs10852936	38031714	*ORMDL3, ZPBP2, GSDMB, IKZF3*	A	0.329	0.260	G	1.03E-10	1.40 (1.26 - 1.54)
17q12	rs2872507	38040763	*ORMDL3, ZPBP2, GSDMB, IKZF3*	A	0.329	0.260	G	1.02E-10	1.39 (1.26 - 1.54)
17q12	rs12950743	38049233	*ORMDL3, ZPBP2, GSDMB, IKZF3*	G	0.328	0.262	A	3.74E-10	1.38 (1.25 - 1.53)
17q12	rs8067378	38051348	*ORMDL3, ZPBP2, GSDMB, IKZF3*	G	0.330	0.262	A	1.30E-10	1.39 (1.26 - 1.54)
17q12	rs2305480	38062196	*ORMDL3, ZPBP2, GSDMB, IKZF3*	A	0.328	0.260	G	1.96E-10	1.39 (1.25 - 1.54)
17q12	rs7216389	38069949	*ORMDL3, ZPBP2, GSDMB, IKZF3*	G	0.328	0.267	A	8.81E-09	1.34 (1.22 - 1.49)
17q12	rs7219923	38074518	*ORMDL3, ZPBP2, GSDMB, IKZF3*	G	0.329	0.268	A	8.18E-09	1.34 (1.22 - 1.49)
17q12	rs7224129	38075426	*ORMDL3, ZPBP2, GSDMB, IKZF3*	G	0.337	0.275	A	1.02E-08	1.34 (1.21 - 1.48)
17q12	rs12603332	38082807	*ORMDL3, ZPBP2, GSDMB, IKZF3*	A	0.333	0.274	G	3.81E-08	1.33 (1.20 - 1.47)

17q12	rs3744246	38084350	*ORMDL3, ZPBP2, GSDMB, IKZF3*	A	0.316	0.263	G	5.27E-07	1.30 (1.17 - 1.44)
17q12	rs4795405	38088417	*ORMDL3, ZPBP2, GSDMB, IKZF3*	A	0.333	0.273	G	2.85E-08	1.33 (1.20 - 1.47)
19p13.3	rs350146	931523	*ARID3A*	A	0.519	0.459	G	5.02E-07	1.27 (1.16 - 1.40)
19p13.3	rs62132345	937480	*ARID3A*	A	0.163	0.211	G	4.39E-07	0.73 (0.64 - 0.82)
19p13.3	rs10414193	939697	*ARID3A*	G	0.403	0.471	A	1.09E-08	0.76 (0.69 - 0.83)
19p13.3	rs10415976	941603	*ARID3A*	G	0.414	0.486	A	1.06E-09	0.75 (0.68 - 0.82)
19p13.3	rs2238573	945013	*ARID3A*	A	0.223	0.280	G	7.89E-08	0.74 (0.66 - 0.83)
22q13.1	rs742402	39659487	*PDGFB, RPL3, SYNGR1*	G	0.128	0.171	A	1.29E-06	0.71 (0.62 - 0.82)
22q13.1	rs742403	39660190	*PDGFB, RPL3, SYNGR1*	A	0.121	0.158	G	1.37E-05	0.73 (0.64 - 0.84)
22q13.1	rs5757596	39668961	*PDGFB, RPL3, SYNGR1*	C	0.120	0.153	A	9.65E-05	0.76 (0.66 - 0.87)
22q13.1	rs11089939	39680957	*PDGFB, RPL3, SYNGR1*	A	0.147	0.185	G	3.19E-05	0.76 (0.67 - 0.87)
22q13.1	rs126228	39681436	*PDGFB, RPL3, SYNGR1*	A	0.110	0.147	G	5.41E-06	0.71 (0.62 - 0.83)
22q13.1	rs137594	39691221	*PDGFB, RPL3, SYNGR1*	A	0.206	0.251	G	1.09E-05	0.77 (0.69 - 0.87)
22q13.1	rs137602	39693408	*PDGFB, RPL3, SYNGR1*	A	0.206	0.249	G	2.43E-05	0.78 (0.70 - 0.88)
22q13.1	rs137603	39694225	*PDGFB, RPL3, SYNGR1*	C	0.109	0.152	A	2.06E-07	0.68 (0.59 - 0.79)
22q13.1	rs971964	39702473	*PDGFB, RPL3, SYNGR1*	G	0.217	0.263	A	1.17E-05	0.78 (0.70 - 0.87)
22q13.1	rs5757611	39708357	*PDGFB, RPL3, SYNGR1*	G	0.210	0.259	A	1.55E-06	0.76 (0.68 - 0.85)
22q13.1	rs137621	39710244	*PDGFB, RPL3, SYNGR1*	A	0.082	0.116	G	4.35E-06	0.68 (0.58 - 0.80)
22q13.1	rs2072872	39713128	*PDGFB, RPL3, SYNGR1*	A	0.084	0.118	G	3.98E-06	0.68 (0.58 - 0.80)
22q13.1	rs5757613	39715262	*PDGFB, RPL3, SYNGR1*	G	0.117	0.154	A	1.19E-05	0.73 (0.63 - 0.84)
22q13.1	rs12627970	39721745	*PDGFB, RPL3, SYNGR1*	A	0.206	0.256	G	1.31E-06	0.76 (0.67 - 0.85)
22q13.1	rs137672	39737094	*PDGFB, RPL3, SYNGR1*	A	0.085	0.117	G	1.52E-05	0.70 (0.59 - 0.82)
22q13.1	rs2069235	39747780	*PDGFB, RPL3, SYNGR1*	G	0.178	0.230	A	1.67E-07	0.73 (0.64 - 0.82)

Supplementary Table 6: SNPs selected for the replication study and combined P values

Chr	SNP	Minor Allele	Position* (bp)	Genes in the Region	GWAS cohort				Replication cohort				Combined samples	
					MAF (case)	MAF (control)	*P* Value	OR (95% CI)	MAF (case)	MAF (control)	*P* Value	OR (95% CI)	*P* Value	OR (95% CI)
6p21	rs9268644	A	32408044	*HLA-DRA*	0.098	0.177	2.41×10^{-19}	0.51 (0.44-0.59)	0.094	0.169	5.84×10^{-14}	0.51 (0.43-0.61)	7.83×10^{-31}	0.51 (0.45-0.57)
6p21	rs9501251	G	33049663	*HLA-DPB1*	0.082	0.044	8.17×10^{-13}	1.94 (1.61-2.33)	0.091	0.045	6.18×10^{-12}	2.11 (1.70-2.62)	2.10×10^{-22}	2.01 (1.76-2.32)
9q32	rs4979467	A	117630043	*TNFSF15,TNFSF8*	0.399	0.322	8.28×10^{-12}	1.40 (1.27-1.54)	0.427	0.230	1.87×10^{-21}	1.74 (1.55-1.95)	1.22×10^{-29}	1.53 (1.42-1.64)
17q12	rs9635726	G	38020141	multiple genes	0.382	0.309	6.42×10^{-11}	1.38 (1.25-1.52)	0.376	0.309	4.24×10^{-7}	1.35 (1.20-1.52)	1.62×10^{-16}	1.37 (1.27-1.48)
11q23.3	rs77871618	A	118733624	*DDX6, CXCR5*	0.225	0.158	9.12×10^{-14}	1.55 (1.38-1.74)	0.202	0.175	1.20×10^{-2}	1.20 (1.04-1.38)	2.56×10^{-13}	1.40 (1.28-1.53)
3q13.33	rs3732421	G	119150089	*CD80*	0.271	0.332	3.76×10^{-8}	0.75 (0.67-0.83)	0.261	0.324	1.51×10^{-6}	0.74 (0.65-0.83)	3.10×10^{-13}	0.74 (0.68-0.80)
2q32.3	rs10168266	A	191935804	*STAT1,STAT4*	0.397	0.336	5.82×10^{-8}	1.31 (1.19-1.44)	0.393	0.328	1.26×10^{-6}	1.33 (1.18-1.49)	3.95×10^{-13}	1.31 (1.22-1.41)
3q25.33	rs582537	C	159710098	*IL12A*	0.234	0.291	6.55×10^{-8}	0.74 (0.66-0.83)	0.237	0.287	6.83×10^{-5}	0.77 (0.68-0.88)	2.36×10^{-11}	0.75 (0.69-0.82)
4q24	rs1598856	G	103446115	*NF-kB1*	0.536	0.474	1.83×10^{-7}	1.28 (1.17-1.41)	0.527	0.474	2.07×10^{-4}	1.23 (1.10-1.39)	1.80×10^{-10}	1.26 (1.17-1.35)
12p13.31	rs4149576	A	6449115	*TNFRSF1A*	0.148	0.113	1.11×10^{-5}	1.35 (1.18-1.55)	0.147	0.110	7.27×10^{-5}	1.39 (1.18-1.63)	3.81×10^{-9}	1.37 (1.23-1.52)
22q13.1	rs137603	C	39694225	*RPL3,SYNGR1*	0.109	0.152	2.06×10^{-7}	0.68 (0.59-0.79)	0.119	0.144	1.10×10^{-2}	0.81 (0.68-0.95)	2.67×10^{-8}	0.73 (0.65-0.81)
16p12.1	rs2189521	G	27413566	*IL4R, IL21R*	0.242	0.306	5.40×10^{-9}	0.73 (0.65-0.81)	0.246	0.320	9.87×10^{-9}	0.69 (0.61-0.79)	4.00×10^{-16}	0.71 (0.66-0.78)
16p12.1	rs10852316	A	27398555	*IL4R, IL21R*	0.379	0.442	8.83×10^{-8}	0.77 (0.70-0.85)	0.374	0.444	5.59×10^{-7}	0.75 (0.67-0.84)	2.76×10^{-13}	0.76 (0.71-0.82)
2q33.2	rs4675369	G	204643194	*CD28, CTLA4*	0.526	0.448	6.56×10^{-11}	1.37 (1.24-1.50)	0.505	0.452	1.65×10^{-4}	1.24 (1.11-1.38)	1.38×10^{-13}	1.31 (1.22-1.41)
2q33.2	rs7599230	G	204648661	*CD28, CTLA4*	0.471	0.408	9.08×10^{-8}	1.29 (1.18-1.42)	0.459	0.411	5.96×10^{-4}	1.22 (1.09-1.36)	3.30×10^{-10}	1.26 (1.18-1.36)
4q27	rs925550	A	123588526	*IL21*	0.428	0.370	6.21×10^{-7}	1.27 (1.16-1.40)	0.427	0.353	7.44×10^{-8}	1.37 (1.22-1.53)	3.87×10^{-13}	1.31 (1.21-1.40)
4q27	rs17005934	G	123549699	*IL21*	0.408	0.352	1.42×10^{-6}	1.27 (1.15-1.39)	0.428	0.362	1.25×10^{-6}	1.32 (1.18-1.48)	1.06×10^{-11}	1.29 (1.21-1.39)
1p13.1	rs2300747	A	117104215	*CD58*	0.481	0.416	4.54×10^{-8}	1.30 (1.18-1.43)	0.475	0.412	8.02×10^{-6}	1.29 (1.15-1.44)	1.84×10^{-12}	1.29 (1.20-1.39)
1p13.1	rs10924106	A	117053745	*CD58*	0.509	0.449	4.58×10^{-7}	1.27 (1.16-1.40)	0.508	0.446	1.10×10^{-5}	1.28 (1.15-1.43)	2.40×10^{-11}	1.28 (1.19-1.37)
19p13.3	rs10415976	G	941603	*ARID3A*	0.414	0.486	1.06×10^{-9}	0.75 (0.68-0.82)	0.439	0.489	3.51×10^{-4}	0.82 (0.73-0.91)	3.61×10^{-12}	0.77 (0.72-0.84)
19p13.3	rs10414193	G	939697	*ARID3A*	0.403	0.471	1.09×10^{-8}	0.76 (0.69-0.83)	0.426	0.474	6.84×10^{-4}	0.82 (0.74-0.92)	5.80×10^{-11}	0.79 (0.73-0.85)
15q25.1	rs11556218	C	81598269	*IL16*	0.233	0.189	3.03×10^{-6}	1.31 (1.17-1.46)	0.226	0.188	6.94×10^{-4}	1.26 (1.10-1.45)	8.99×10^{-9}	1.29 (1.18-1.41)
16q21	rs2550374	A	58254448	*CSNK2A2, CCDC113*	0.424	0.484	4.19×10^{-7}	0.78 (0.71-0.86)	0.446	0.486	5.26×10^{-3}	0.85 (0.76-0.95)	1.51×10^{-8}	0.81 (0.76-0.87)
6p25.2	rs4305775	C	3296997	*SLC22A23*	0.227	0.286	2.77×10^{-8}	0.73 (0.66-0.82)	0.270	0.291	0.097	0.90 (0.80-1.02)	1.52×10^{-7}	0.80 (0.74-0.88)
6p25.2	rs9378359	A	3297021	*SLC22A23*	0.176	0.225	4.69×10^{-7}	0.73 (0.65-0.83)	0.204	0.230	0.027	0.86 (0.75-0.98)	1.78×10^{-7}	0.79 (0.72-0.87)
6q27	rs6456156	A	167522300	*FGFR10P, CCR6*	0.425	0.488	2.04×10^{-7}	0.78 (0.71-0.86)	0.463	0.485	0.128	0.92 (0.82-1.03)	6.26×10^{-7}	0.83 (0.78-0.90)
4q34.1	rs6856421	A	175556816	*GLRA3*	0.308	0.257	1.68×10^{-6}	1.28 (1.16-1.42)	0.271	0.249	0.083	1.12 (0.98-1.27)	1.59×10^{-6}	1.22 (1.11-1.30)
6q24.2	rs197465	A	143080039	*HIVEP2*	0.543	0.485	8.75×10^{-7}	1.27 (1.15-1.39)	0.500	0.487	0.350	1.05 (0.94-1.18)	1.37×10^{-5}	1.17 (1.09-1.25)
22q11.23	rs5760096	G	24247310	*MIF*	0.394	0.339	1.41×10^{-6}	1.27 (1.15-1.40)	0.356	0.344	0.376	1.05 (0.94-1.18)	1.94×10^{-5}	1.18 (1.09-1.26)
22q11.23	rs5751763	T	24247793	*MIF*	0.393	0.337	6.51×10^{-7}	1.28 (1.16-1.41)	0.345	0.344	0.958	1.00 (0.89-1.13)	1.17×10^{-4}	1.16 (1.07-1.24)
3p24.3	rs6765957	A	17135597	*PLCL2*	0.347	0.401	3.65×10^{-6}	0.79 (0.72-0.87)	0.379	0.375	0.787	1.02 (0.91-1.14)	8.31×10^{-4}	0.88 (0.81-0.94)
22q12.2	rs2267168	C	31643480	*LIMK2, PATZ1*	0.418	0.364	2.03×10^{-6}	1.26 (1.15-1.39)	0.365	0.375	0.502	0.96 (0.86-1.08)	1.30×10^{-3}	1.13 (1.04-1.21)
22q12.2	rs5753572	A	31754044	*LIMK2, PATZ1*	0.415	0.361	2.54×10^{-6}	1.26 (1.14-1.38)	0.357	0.372	0.285	0.94 (0.84-1.05)	3.30×10^{-3}	1.12 (1.03-1.19)
11q23.1	rs1944918	A	111261995	POU2AF1	0.421	0.473	1.47×10^{-5}	0.81 (0.74-0.89)	0.462	0.472	0.514	0.96 (0.86-1.08)	2.04×10^{-4}	0.87 (0.82-0.94)

* Sequence position was annotated based on the GRCh37/hg19 assembly. Chr., chromosome; MAF, minor allele frequency. P value is based on additive model; OR, odds ratio, is calculated for minor allele

Supplementary Table 7: SNPs genotyped and imputed in six new loci associated with PBC with p value <1×10^{-4}

CHR	SNP	BP*	Allele 1	MAF (case)	MAF (control)	Allele 2	*P* value#	OR (95% CI)	Gentyping	Annotation	Locus/Gene
1	rs956184	117040622	G	0.491	0.550	C	6.31E-07	0.79 (0.72-0.87)	imputed	3'downstream	*CD58*
1	rs3850814	117043044	T	0.480	0.424	C	2.02E-06	1.26 (1.14-1.38)	imputed	3'downstream	*CD58*
1	rs9651076	117043302	A	0.480	0.424	G	2.02E-06	1.26 (1.14-1.38)	imputed	3'downstream	*CD58*
1	rs1109190	117046263	C	0.491	0.550	G	6.31E-07	0.79 (0.72-0.87)	imputed	3'downstream	*CD58*
1	rs10754443	117048732	T	0.491	0.551	A	4.82E-07	0.79 (0.72-0.86)	imputed	3'downstream	*CD58*
1	rs10924104	117048903	G	0.482	0.427	A	2.59E-06	1.25 (1.14-1.38)	imputed	3'downstream	*CD58*
1	rs10754444	117049676	A	0.4821	0.4266	G	2.687E-06	1.25 (1.14-1.38)	genotyped	3'downstream	*CD58*
1	rs10737769	117050164	T	0.491	0.551	A	4.82E-07	0.79 (0.72-0.86)	imputed	3'downstream	*CD58*
1	rs10924106	117053745	C	0.491	0.550	T	5.39E-07	0.79 (0.72-0.86)	genotyped	3'downstream	*CD58*
1	rs12031061	117054011	A	0.491	0.550	G	5.39E-07	0.79 (0.72-0.86)	imputed	3'downstream	*CD58*
1	rs4468196	117054221	A	0.493	0.551	G	1.41E-06	0.79 (0.72-0.87)	imputed	3'downstream	*CD58*
1	rs10802188	117054887	G	0.491	0.551	A	8.26E-07	0.79 (0.71-0.87)	imputed	3'downstream	*CD58*
1	rs10802190	117061384	T	0.490	0.550	A	4.49E-07	0.79 (0.72-0.86)	imputed	3'UTR	*CD58*
1	rs10924108	117062474	C	0.492	0.551	T	4.65E-07	0.79 (0.72-0.86)	genotyped	intronic	*CD58*
1	rs10802191	117065083	A	0.491	0.550	T	5.39E-07	0.79 (0.72-0.86)	imputed	intronic	*CD58*
1	rs1414275	117066907	G	0.486	0.551	A	3.76E-08	0.77 (0.7-0.84)	genotyped	intronic	*CD58*
1	rs11588376	117072524	C	0.486	0.551	T	3.76E-08	0.77 (0.7-0.84)	imputed	intronic	*CD58*
1	rs1034919	117075505	C	0.486	0.551	G	3.76E-08	0.77 (0.7-0.84)	imputed	intronic	*CD58*
1	rs1034920	117076399	C	0.492	0.551	T	4.65E-07	0.79 (0.72-0.86)	imputed	intronic	*CD58*
1	rs1034921	117076403	A	0.492	0.551	G	4.65E-07	0.79 (0.72-0.86)	imputed	intronic	*CD58*
1	rs10802192	117076461	C	0.492	0.551	T	4.65E-07	0.79 (0.72-0.86)	imputed	intronic	*CD58*
1	rs1016140	117076547	T	0.492	0.551	G	4.65E-07	0.79 (0.72-0.86)	imputed	intronic	*CD58*
1	rs10754445	117077459	T	0.486	0.551	A	3.76E-08	0.77 (0.7-0.84)	imputed	intronic	*CD58*
1	rs10924109	117079262	C	0.493	0.551	T	1.12E-06	0.79 (0.72-0.87)	imputed	intronic	*CD58*
1	rs6677309	117080166	A	0.480	0.423	C	1.76E-06	1.26 (1.14-1.38)	genotyped	intronic	*CD58*
1	rs758518	117081810	G	0.493	0.551	A	1.12E-06	0.79 (0.72-0.87)	genotyped	intronic	*CD58*
1	rs2300746	117082183	G	0.486	0.551	C	3.76E-08	0.77 (0.7-0.84)	imputed	intronic	*CD58*

1	rs12141411	117084410	A	0.492	0.551	G	4.65E-07	0.79 (0.72-0.86)	imputed	intronic	*CD58*
1	rs12044773	117087502	A	0.490	0.548	C	1.15E-06	0.79 (0.72-0.87)	imputed	intronic	*CD58*
1	rs12044852	117087779	A	0.490	0.548	C	1.23E-06	0.79 (0.72-0.87)	genotyped	intronic	*CD58*
1	rs12038673	117088636	A	0.490	0.548	G	1.23E-06	0.79 (0.72-0.87)	imputed	intronic	*CD58*
1	rs35275493	117095502	AT	0.490	0.548	A	1.23E-06	0.79 (0.72-0.87)	imputed	intronic	*CD58*
1	rs1335532	117100957	A	0.480	0.423	G	1.76E-06	1.26 (1.14-1.38)	imputed	intronic	*CD58*
1	rs1414273	117102649	C	0.480	0.423	T	1.76E-06	1.26 (1.14-1.38)	imputed	intronic	*CD58*
1	rs2300747	117104215	A	0.481	0.416	G	3.38E-08	1.3 (1.19-1.43)	genotyped	intronic	*CD58*
2	rs3181094	204569717	G	0.319	0.371	T	9.75E-06	0.79 (0.71-0.88)	imputed	promoter region	*CD28*
2	rs1879877	204570000	G	0.331	0.384	T	4.70E-06	0.79 (0.72-0.88)	genotyped	promoter region	*CD28*
2	rs1181388	204575951	G	0.404	0.459	A	4.37E-06	0.8 (0.73-0.88)	genotyped	intronic	*CD28*
2	rs12468949	204604603	G	0.324	0.383	A	3.30E-07	0.77 (0.7-0.85)	imputed	3'downstream	*CD28*
2	rs6435203	204611195	A	0.324	0.383	G	3.30E-07	0.77 (0.7-0.85)	imputed	3'downstream	*CD28*
2	rs1961361	204619397	C	0.319	0.379	T	2.27E-07	0.77 (0.7-0.85)	imputed	intergenic	*CD28-CTLA4*
2	rs2352407	204623245	A	0.319	0.379	C	2.27E-07	0.77 (0.7-0.85)	imputed	intergenic	*CD28-CTLA4*
2	rs73991099	204623901	T	0.571	0.524	C	9.10E-05	1.21 (1.1-1.33)	imputed	intergenic	*CD28-CTLA4*
2	rs10186048	204624056	A	0.319	0.379	G	2.27E-07	0.77 (0.7-0.85)	imputed	intergenic	*CD28-CTLA4*
2	rs73991101	204626795	A	0.444	0.379	T	6.44E-08	1.31 (1.19-1.45)	imputed	intergenic	*CD28-CTLA4*
2	rs10932018	204627407	A	0.408	0.483	G	4.00E-10	0.74 (0.67-0.81)	imputed	intergenic	*CD28-CTLA4*
2	rs12467195	204628328	G	0.408	0.483	A	4.00E-10	0.74 (0.67-0.81)	imputed	intergenic	*CD28-CTLA4*
2	rs10932019	204628810	A	0.408	0.483	G	4.00E-10	0.74 (0.67-0.81)	imputed	intergenic	*CD28-CTLA4*
2	rs1974996	204629421	T	0.408	0.483	C	4.00E-10	0.74 (0.67-0.81)	imputed	intergenic	*CD28-CTLA4*
2	rs7420136	204632071	T	0.408	0.483	C	4.00E-10	0.74 (0.67-0.81)	imputed	intergenic	*CD28-CTLA4*
2	rs10210464	204632810	C	0.408	0.483	T	4.00E-10	0.74 (0.67-0.81)	imputed	intergenic	*CD28-CTLA4*
2	rs231387	204632931	G	0.408	0.483	A	4.00E-10	0.74 (0.67-0.81)	imputed	intergenic	*CD28-CTLA4*
2	rs10932020	204633156	A	0.408	0.483	G	3.24E-10	0.74 (0.67-0.81)	genotyped	intergenic	*CD28-CTLA4*
2	rs10932021	204633260	A	0.408	0.483	C	3.24E-10	0.74 (0.67-0.81)	imputed	intergenic	*CD28-CTLA4*
2	rs73993004	204634674	T	0.471	0.408	A	8.65E-08	1.29 (1.18-1.42)	imputed	intergenic	*CD28-CTLA4*
2	rs231390	204634767	A	0.312	0.375	G	3.80E-08	0.76 (0.68-0.84)	genotyped	intergenic	*CD28-CTLA4*
2	rs76971408	204635376	G	0.471	0.408	A	8.65E-08	1.29 (1.18-1.42)	imputed	intergenic	*CD28-CTLA4*
2	rs74267897	204635447	T	0.471	0.408	C	8.65E-08	1.29 (1.18-1.42)	imputed	intergenic	*CD28-CTLA4*

2	rs6732274	204637162	G	0.540	0.465	T	4.91E-10	1.35 (1.23-1.49)	imputed	intergenic	*CD28-CTLA4*
2	rs4673268	204639764	A	0.470	0.407	C	8.57E-08	1.29 (1.18-1.42)	genotyped	intergenic	*CD28-CTLA4*
2	rs62184023	204641761	T	0.526	0.448	C	4.02E-11	1.37 (1.25-1.51)	imputed	intergenic	*CD28-CTLA4*
2	rs4675369	204643194	G	0.526	0.448	A	5.81E-11	1.37 (1.24-1.5)	genotyped	intergenic	*CD28-CTLA4*
2	rs4675370	204646499	G	0.526	0.448	C	5.39E-11	1.37 (1.25-1.5)	imputed	intergenic	*CD28-CTLA4*
2	rs7599230	204648661	C	0.471	0.408	T	8.65E-08	1.29 (1.18-1.42)	genotyped	intergenic	*CD28-CTLA4*
2	rs76512872	204649395	C	0.471	0.408	T	8.65E-08	1.29 (1.18-1.42)	imputed	intergenic	*CD28-CTLA4*
2	rs3096867	204650863	A	0.471	0.408	T	8.65E-08	1.29 (1.18-1.42)	imputed	intergenic	*CD28-CTLA4*
2	rs74267901	204653860	A	0.464	0.400	G	4.28E-08	1.3 (1.19-1.43)	imputed	intergenic	*CD28-CTLA4*
2	rs16840125	204655597	T	0.471	0.408	A	8.65E-08	1.29 (1.18-1.42)	imputed	intergenic	*CD28-CTLA4*
2	rs3116507	204661524	A	0.392	0.459	G	1.45E-08	0.76 (0.69-0.83)	imputed	intergenic	*CD28-CTLA4*
2	rs7591187	204676238	T	0.397	0.469	C	1.40E-09	0.75 (0.68-0.82)	genotyped	intergenic	*CD28-CTLA4*
2	rs12616405	204683511	C	0.393	0.452	G	6.82E-07	0.79 (0.71-0.86)	imputed	intergenic	*CD28-CTLA4*
2	rs1018361	204684835	T	0.393	0.452	C	6.43E-07	0.79 (0.71-0.86)	genotyped	intergenic	*CD28-CTLA4*
2	rs231747	204688432	T	0.462	0.410	G	9.91E-06	1.24 (1.13-1.36)	genotyped	intergenic	*CD28-CTLA4*
2	rs231735	204693876	G	0.157	0.193	T	9.92E-05	0.78 (0.69-0.88)	imputed	intergenic	*CD28-CTLA4*
2	rs231734	204694280	G	0.157	0.193	A	9.92E-05	0.78 (0.69-0.88)	imputed	intergenic	*CD28-CTLA4*
2	rs231733	204694465	A	0.157	0.193	G	9.92E-05	0.78 (0.69-0.88)	imputed	intergenic	*CD28-CTLA4*
2	rs3116514	204694784	G	0.157	0.193	A	9.92E-05	0.78 (0.69-0.88)	imputed	intergenic	*CD28-CTLA4*
2	rs231789	204699952	C	0.162	0.203	T	3.09E-05	0.76 (0.67-0.87)	imputed	intergenic	*CD28-CTLA4*
2	rs231790	204700574	G	0.162	0.203	T	3.09E-05	0.76 (0.67-0.87)	imputed	intergenic	*CD28-CTLA4*
2	rs231791	204701865	C	0.162	0.203	A	3.09E-05	0.76 (0.67-0.87)	imputed	intergenic	*CD28-CTLA4*
2	rs231792	204702249	A	0.162	0.203	G	3.09E-05	0.76 (0.67-0.87)	imputed	intergenic	*CD28-CTLA4*
2	rs231796	204704702	G	0.162	0.203	A	3.09E-05	0.76 (0.67-0.87)	imputed	intergenic	*CD28-CTLA4*
2	rs2162606	204705157	C	0.162	0.202	G	2.84E-05	0.76 (0.67-0.86)	imputed	intergenic	*CD28-CTLA4*
2	rs231797	204706107	A	0.162	0.202	G	2.84E-05	0.76 (0.67-0.86)	imputed	intergenic	*CD28-CTLA4*
2	rs231799	204707417	C	0.162	0.202	T	2.84E-05	0.76 (0.67-0.86)	imputed	intergenic	*CD28-CTLA4*
2	rs231800	204707585	G	0.160	0.199	C	3.83E-05	0.76 (0.67-0.87)	imputed	intergenic	*CD28-CTLA4*
2	rs78707218	204710197	A	0.466	0.413	G	1.23E-05	1.24 (1.13-1.36)	genotyped	intergenic	*CD28-CTLA4*
2	rs11571292	204720139	G	0.290	0.337	A	2.57E-05	0.8 (0.72-0.89)	imputed	intergenic	*CD28-CTLA4*
2	rs231763	204720895	T	0.286	0.333	G	2.49E-05	0.8 (0.72-0.89)	imputed	intergenic	*CD28-CTLA4*

2	rs11571291	204721132	C	0.157	0.194	T	7.89E-05	0.78 (0.68-0.88)	genotyped	intergenic	*CD28-CTLA4*
2	rs1024161	204721752	C	0.286	0.334	T	1.98E-05	0.8 (0.72-0.89)	imputed	intergenic	*CD28-CTLA4*
2	rs1024162	204722159	T	0.157	0.193	A	7.93E-05	0.78 (0.68-0.88)	imputed	intergenic	*CD28-CTLA4*
2	rs926169	204722752	G	0.290	0.337	T	2.57E-05	0.8 (0.72-0.89)	imputed	intergenic	*CD28-CTLA4*
2	rs231764	204723541	C	0.285	0.333	T	2.17E-05	0.8 (0.72-0.89)	imputed	intergenic	*CD28-CTLA4*
2	rs1427679	204723750	A	0.286	0.334	G	2.13E-05	0.8 (0.72-0.89)	imputed	intergenic	*CD28-CTLA4*
2	rs231770	204729153	C	0.285	0.333	T	2.17E-05	0.8 (0.72-0.89)	imputed	promoter region	*CTLA4*
2	rs1427680	204729795	A	0.290	0.338	G	2.36E-05	0.8 (0.72-0.89)	imputed	promoter region	*CTLA4*
2	rs736611	204730465	T	0.290	0.338	C	2.36E-05	0.8 (0.72-0.89)	imputed	promoter region	*CTLA4*
2	rs11571315	204730901	C	0.286	0.334	T	2.10E-05	0.8 (0.72-0.89)	genotyped	promoter region	*CTLA4*
2	rs733618	204730944	C	0.447	0.399	T	3.97E-05	1.22 (1.11-1.34)	genotyped	promoter region	*CTLA4*
2	rs231775	204732714	A	0.286	0.334	G	1.50E-05	0.8 (0.72-0.88)	genotyped	coding variant, T17A	*CTLA4*
2	rs231779	204734487	C	0.285	0.333	T	2.17E-05	0.8 (0.72-0.89)	imputed	intronic	*CTLA4*
2	rs231723	204739781	A	0.286	0.333	G	2.49E-05	0.8 (0.72-0.89)	imputed	3'downstream	*CTLA4*
2	rs231724	204739823	A	0.286	0.333	G	2.49E-05	0.8 (0.72-0.89)	imputed	3'downstream	*CTLA4*
2	rs2307982	204740446	CAAGG	0.318	0.366	C	6.38E-05	0.81 (0.73-0.9)	imputed	3'downstream	*CTLA4*
2	rs231725	204740675	G	0.318	0.366	A	6.38E-05	0.81 (0.73-0.9)	imputed	3'downstream	*CTLA4*
2	rs1427676	204741166	T	0.318	0.366	C	5.95E-05	0.81 (0.73-0.9)	imputed	3'downstream	*CTLA4*
2	rs231727	204741550	G	0.318	0.366	A	5.95E-05	0.81 (0.73-0.9)	imputed	3'downstream	*CTLA4*
2	rs231729	204743801	T	0.318	0.366	A	5.95E-05	0.81 (0.73-0.9)	imputed	3'downstream	*CTLA4*
4	rs4833824	123325183	A	0.425	0.378	G	6.31E-05	1.21 (1.1-1.34)	imputed	intronic	*ADAD1*
4	rs4833825	123340114	G	0.425	0.378	A	6.31E-05	1.21 (1.1-1.34)	imputed	intronic	*ADAD1*
4	rs147174273	123362607	G	0.425	0.378	T	6.31E-05	1.21 (1.1-1.34)	imputed	intergenic	*ADAD1-IL2*
4	rs10857091	123365933	C	0.425	0.378	T	6.31E-05	1.21 (1.1-1.34)	imputed	3'downstream	*IL2*
4	rs45628031	123386701	A	0.425	0.378	G	6.31E-05	1.21 (1.1-1.34)	imputed	5'upstream	*IL2*
4	rs10857092	123389219	A	0.425	0.378	G	6.89E-05	1.21 (1.1-1.33)	genotyped	intergenic	*IL2-IL21*
4	rs11098661	123433673	C	0.425	0.378	A	6.51E-05	1.21 (1.1-1.33)	imputed	intergenic	*IL2-IL21*
4	rs6828117	123434242	A	0.400	0.350	G	2.53E-05	1.24 (1.12-1.37)	imputed	intergenic	*IL2-IL21*
4	rs58207441	123477175	G	0.425	0.378	C	6.89E-05	1.21 (1.1-1.33)	imputed	intergenic	*IL2-IL21*
4	rs17005934	123549699	C	0.407	0.352	T	1.86E-06	1.26 (1.15-1.39)	genotyped	5'upstream	*IL21*

4	rs17005953	123556241	T	0.404	0.350	C	1.86E-06	1.26 (1.15-1.39)	imputed	5'upstream	*IL21*
4	rs12511287	123578531	A	0.505	0.455	T	2.06E-05	1.23 (1.12-1.35)	imputed	5'upstream	*IL21*
4	rs6825988	123582081	A	0.505	0.455	G	2.06E-05	1.23 (1.12-1.35)	imputed	5'upstream	*IL21*
4	rs2036982	123584946	C	0.505	0.455	T	2.06E-05	1.23 (1.12-1.35)	imputed	5'upstream	*IL21*
4	rs925550	123588526	A	0.4277	0.37	C	5.21E-07	1.27 (1.16-1.4)	genotyped	5'upstream	*IL21*
4	rs1913191	123601514	T	0.505	0.455	A	2.06E-05	1.23 (1.12-1.35)	imputed	5'upstream	*IL21*
4	rs6836610	123601697	A	0.505	0.455	G	2.06E-05	1.23 (1.12-1.35)	genotyped	5'upstream	*IL21*
4	rs309401	123616211	G	0.409	0.361	A	3.20E-05	1.23 (1.11-1.35)	imputed	5'upstream	*IL21*
4	rs145880173	123618202	A	0.355	0.307	AAG	1.76E-05	1.24 (1.13-1.37)	imputed	5'upstream	*IL21*
4	rs746550	123619689	T	0.361	0.314	C	2.35E-05	1.24 (1.12-1.36)	imputed	5'upstream	*IL21*
4	rs1026157	123623893	G	0.360	0.314	T	3.78E-05	1.23 (1.11-1.36)	genotyped	5'upstream	*IL21*
4	rs62322204	123629089	T	0.360	0.314	C	3.78E-05	1.23 (1.11-1.36)	imputed	5'upstream	*IL21*
4	rs416556	123629427	C	0.411	0.362	A	2.89E-05	1.23 (1.11-1.35)	genotyped	5'upstream	*IL21*
4	rs17006053	123629661	A	0.360	0.314	C	3.78E-05	1.23 (1.11-1.36)	imputed	5'upstream	*IL21*
4	rs2893009	123629927	C	0.413	0.365	T	2.91E-05	1.23 (1.11-1.35)	imputed	5'upstream	*IL21*
4	rs147384934	123630024	C	0.360	0.314	CAATT	3.78E-05	1.23 (1.11-1.36)	imputed	5'upstream	*IL21*
4	rs427081	123630743	A	0.411	0.362	G	2.89E-05	1.23 (1.11-1.35)	imputed	5'upstream	*IL21*
4	rs1512974	123631308	G	0.413	0.365	A	2.81E-05	1.23 (1.11-1.35)	genotyped	5'upstream	*IL21*
4	rs368797	123635211	T	0.410	0.360	C	1.17E-05	1.24 (1.13-1.36)	imputed	intergenic	
4	rs33995758	123635653	T	0.410	0.360	TG	1.22E-05	1.24 (1.13-1.36)	imputed	intergenic	
4	rs381910	123635717	A	0.410	0.360	T	1.28E-05	1.24 (1.12-1.36)	imputed	intergenic	
4	rs370250	123635802	C	0.409	0.360	T	2.59E-05	1.23 (1.12-1.35)	imputed	intergenic	
4	rs12054579	123639347	T	0.413	0.365	C	2.81E-05	1.23 (1.11-1.35)	imputed	intergenic	
4	rs1900969	123640314	G	0.413	0.365	C	2.81E-05	1.23 (1.11-1.35)	imputed	intergenic	
4	rs309343	123643385	G	0.410	0.360	A	1.17E-05	1.24 (1.13-1.36)	imputed	intergenic	
4	rs309348	123646391	A	0.410	0.360	C	1.17E-05	1.24 (1.13-1.36)	imputed	5'upstream	*BBS12*
4	rs309350	123647342	C	0.409	0.360	T	2.59E-05	1.23 (1.12-1.35)	imputed	5'upstream	*BBS12*
4	rs6845853	123648601	A	0.410	0.360	G	1.22E-05	1.24 (1.13-1.36)	imputed	5'upstream	*BBS12*
4	rs7693576	123648898	G	0.413	0.365	A	2.81E-05	1.23 (1.11-1.35)	imputed	5'upstream	*BBS12*
4	rs309353	123649223	T	0.411	0.363	C	2.51E-05	1.23 (1.12-1.35)	imputed	5'upstream	*BBS12*
4	rs61025664	123651826	A	0.413	0.365	C	2.81E-05	1.23 (1.11-1.35)	imputed	promoter region	*BBS12*

4	rs12640277	123651839	T	0.413	0.365	C	2.81E-05	1.23 (1.11-1.35)	imputed	promoter region	*BBS12*
4	rs12649836	123651888	G	0.413	0.365	A	2.81E-05	1.23 (1.11-1.35)	imputed	promoter region	*BBS12*
4	rs309357	123653576	G	0.410	0.360	T	1.17E-05	1.24 (1.13-1.36)	imputed	promoter region	*BBS12*
4	rs7658801	123656408	A	0.411	0.362	G	1.79E-05	1.23 (1.12-1.36)	imputed	intronic	*BBS12*
4	rs72671080	123659793	G	0.413	0.364	A	1.77E-05	1.23 (1.12-1.36)	imputed	intronic	*BBS12*
4	rs6833709	123662339	T	0.411	0.362	G	1.79E-05	1.23 (1.12-1.36)	imputed	intronic	*BBS12*
4	rs309370	123664204	G	0.412	0.361	A	1.15E-05	1.24 (1.13-1.36)	genotyped	coding variant,R386Q	*BBS12*
4	rs2292493	123664445	C	0.414	0.363	T	1.38E-05	1.24 (1.12-1.36)	genotyped	coding variant,G466G	*BBS12*
4	rs4833843	123665428	G	0.414	0.365	A	1.90E-05	1.23 (1.12-1.35)	genotyped	3'UTR	*BBS12*
4	rs309386	123665991	C	0.410	0.360	G	1.23E-05	1.24 (1.13-1.36)	imputed	3'UTR	*BBS12*
4	rs7690409	123666757	A	0.413	0.362	G	9.11E-06	1.24 (1.13-1.37)	imputed	3'downstream	*BBS12*
4	rs309385	123666760	G	0.410	0.360	C	1.17E-05	1.24 (1.13-1.36)	imputed	3'downstream	*BBS12*
4	rs12644866	123667604	T	0.413	0.362	A	9.11E-06	1.24 (1.13-1.37)	imputed	3'downstream	*BBS12*
4	rs309384	123668300	T	0.410	0.360	G	1.17E-05	1.24 (1.13-1.36)	imputed	3'downstream	*BBS12*
4	rs4833844	123668544	C	0.413	0.365	T	2.55E-05	1.23 (1.12-1.35)	genotyped	3'downstream	*BBS12*
4	rs309382	123669772	T	0.410	0.360	C	1.17E-05	1.24 (1.13-1.36)	imputed	intergenic	
4	rs78281501	123670559	TTA	0.413	0.365	T	2.55E-05	1.23 (1.12-1.35)	imputed	intergenic	
4	4:123670684	123670684	C	0.413	0.362	CATTA	9.11E-06	1.24 (1.13-1.37)	imputed	intergenic	
4	rs13117125	123670824	T	0.413	0.362	C	9.11E-06	1.24 (1.13-1.37)	imputed	intergenic	
4	rs13122636	123671045	T	0.413	0.362	C	9.11E-06	1.24 (1.13-1.37)	imputed	intergenic	
4	rs309379	123671456	C	0.410	0.360	G	1.17E-05	1.24 (1.13-1.36)	imputed	intergenic	
4	rs6811183	123671551	T	0.413	0.362	G	9.50E-06	1.24 (1.13-1.36)	genotyped	intergenic	
4	rs9990804	123671984	A	0.413	0.362	G	9.50E-06	1.24 (1.13-1.36)	imputed	intergenic	
4	rs13103818	123672516	G	0.413	0.362	A	9.50E-06	1.24 (1.13-1.36)	imputed	intergenic	
4	rs10016979	123673192	G	0.413	0.362	A	9.50E-06	1.24 (1.13-1.36)	imputed	intergenic	
4	rs7690133	123673809	A	0.413	0.362	G	9.50E-06	1.24 (1.13-1.36)	imputed	intergenic	
4	rs7673455	123674034	T	0.413	0.362	C	9.50E-06	1.24 (1.13-1.36)	imputed	intergenic	
4	rs7695379	123674099	C	0.413	0.362	T	9.50E-06	1.24 (1.13-1.36)	imputed	intergenic	
4	rs1849430	123674701	C	0.413	0.362	T	9.50E-06	1.24 (1.13-1.36)	imputed	intergenic	

4	rs1849431	123674762	G	0.420	0.372	A	3.15E-05	1.23 (1.11-1.35)	imputed	intergenic	
4	rs309377	123676353	G	0.410	0.360	A	1.22E-05	1.24 (1.13-1.36)	imputed	intergenic	
4	rs4833255	123678231	C	0.420	0.371	A	3.25E-05	1.23 (1.11-1.35)	imputed	intergenic	
4	rs2034460	123679913	T	0.413	0.362	C	1.18E-05	1.24 (1.13-1.36)	imputed	intergenic	
4	rs309376	123680191	A	0.410	0.360	G	1.22E-05	1.24 (1.13-1.36)	imputed	intergenic	
4	rs35520881	123680633	A	0.414	0.363	AT	1.38E-05	1.24 (1.12-1.36)	imputed	intergenic	
4	rs1849427	123682325	C	0.412	0.362	T	1.40E-05	1.24 (1.12-1.36)	imputed	intergenic	
4	rs309372	123682639	C	0.410	0.360	T	1.22E-05	1.24 (1.13-1.36)	imputed	intergenic	
4	rs11942009	123684916	C	0.412	0.362	T	1.40E-05	1.24 (1.12-1.36)	imputed	intergenic	
4	rs11098672	123686064	G	0.412	0.362	T	1.40E-05	1.24 (1.12-1.36)	imputed	intergenic	
15	rs17875509	81590775	C	0.232	0.188	G	3.15E-06	1.31 (1.17-1.46)	imputed	intronic	*IL16*
15	rs11857713	81591265	T	0.232	0.188	C	4.33E-06	1.3 (1.16-1.46)	genotyped	intronic	*IL16*
15	rs4778636	81591639	A	0.232	0.188	G	3.47E-06	1.31 (1.17-1.46)	genotyped	intronic	*IL16*
15	rs11073001	81592802	G	0.269	0.224	A	9.91E-06	1.27 (1.14-1.42)	genotyped	coding variant, T344T	*IL16*
15	rs11630277	81592891	T	0.269	0.224	C	9.91E-06	1.27 (1.14-1.42)	imputed	intronic	*IL16*
15	rs56859031	81592975	AGT	0.232	0.188	A	3.47E-06	1.31 (1.17-1.46)	imputed	intronic	*IL16*
15	rs57763246	81595827	T	0.233	0.189	C	3.34E-06	1.31 (1.17-1.46)	imputed	intronic	*IL16*
15	rs17875523	81596552	T	0.233	0.189	C	3.34E-06	1.31 (1.17-1.46)	imputed	intronic	*IL16*
15	rs3898677	81596562	C	0.266	0.221	T	9.21E-06	1.28 (1.15-1.42)	imputed	intronic	*IL16*
15	rs139879640	81596606	T	0.233	0.189	TCTCA	3.34E-06	1.31 (1.17-1.46)	imputed	intronic	*IL16*
15	rs4577037	81596660	G	0.233	0.189	T	3.34E-06	1.31 (1.17-1.46)	imputed	intronic	*IL16*
15	rs3899547	81597399	G	0.232	0.188	A	3.47E-06	1.31 (1.17-1.46)	imputed	intronic	*IL16*
15	rs17875532	81598110	T	0.233	0.189	C	3.48E-06	1.31 (1.17-1.46)	imputed	intronic	*IL16*
15	rs17875533	81598225	A	0.233	0.189	C	3.48E-06	1.31 (1.17-1.46)	imputed	intronic	*IL16*
15	rs11556218	81598269	G	0.233	0.189	T	3.48E-06	1.31 (1.17-1.46)	genotyped	coding variant, N446K	*IL16*
15	rs1803275	81598416	A	0.233	0.189	G	3.48E-06	1.31 (1.17-1.46)	genotyped	coding variant, R495R	*IL16*
15	rs3926277	81599294	A	0.233	0.189	C	3.48E-06	1.31 (1.17-1.46)	imputed	intronic	*IL16*
15	rs3926278	81599355	G	0.232	0.188	C	3.47E-06	1.31 (1.17-1.46)	imputed	intronic	*IL16*

15	rs3926279	81599443	G	0.232	0.188	A	3.47E-06	1.31 (1.17-1.46)	imputed	intronic	*IL16*
15	rs17875542	81599791	C	0.232	0.188	G	3.47E-06	1.31 (1.17-1.46)	imputed	intronic	*IL16*
15	rs17875543	81599806	T	0.232	0.188	C	3.47E-06	1.31 (1.17-1.46)	imputed	intronic	*IL16*
15	rs7166271	81600020	T	0.232	0.188	C	3.47E-06	1.31 (1.17-1.46)	imputed	intronic	*IL16*
15	rs11325	81601340	T	0.282	0.234	G	3.37E-06	1.28 (1.16-1.43)	genotyped	3'UTR	*IL16*
15	rs4778640	81604131	G	0.253	0.209	A	9.67E-06	1.28 (1.15-1.43)	genotyped	3'UTR	*IL16*
16	rs6498015	27391624	A	0.223	0.268	G	5.14E-05	0.79 (0.7-0.88)	imputed	intergenic	*IL4R-IL21R*
16	rs6498017	27394858	A	0.267	0.321	G	9.63E-07	0.77 (0.69-0.86)	imputed	intergenic	*IL4R-IL21R*
16	rs757374	27395178	T	0.267	0.321	C	9.63E-07	0.77 (0.69-0.86)	imputed	intergenic	*IL4R-IL21R*
16	rs722516	27396461	G	0.379	0.443	A	6.41E-08	0.77 (0.7-0.85)	imputed	intergenic	*IL4R-IL21R*
16	rs722517	27396595	T	0.267	0.321	C	9.63E-07	0.77 (0.69-0.86)	imputed	intergenic	*IL4R-IL21R*
16	rs1107788	27397235	G	0.379	0.443	A	6.41E-08	0.77 (0.7-0.85)	imputed	intergenic	*IL4R-IL21R*
16	rs11074854	27397539	G	0.267	0.321	C	9.63E-07	0.77 (0.69-0.86)	imputed	intergenic	*IL4R-IL21R*
16	rs1859309	27397821	G	0.379	0.443	A	6.41E-08	0.77 (0.7-0.85)	imputed	intergenic	*IL4R-IL21R*
16	rs1859308	27397998	A	0.267	0.321	G	9.63E-07	0.77 (0.69-0.86)	genotyped	intergenic	*IL4R-IL21R*
16	rs1859307	27398024	C	0.379	0.443	T	7.60E-08	0.77 (0.7-0.85)	imputed	intergenic	*IL4R-IL21R*
16	rs1859306	27398094	T	0.357	0.421	C	1.11E-07	0.76 (0.69-0.84)	imputed	intergenic	*IL4R-IL21R*
16	rs11074855	27398390	C	0.379	0.443	T	7.60E-08	0.77 (0.7-0.85)	imputed	intergenic	*IL4R-IL21R*
16	rs10852316	27398555	T	0.380	0.443	G	9.83E-08	0.77 (0.7-0.85)	genotyped	intergenic	*IL4R-IL21R*
16	rs7199163	27399100	T	0.267	0.321	G	9.63E-07	0.77 (0.69-0.86)	imputed	intergenic	*IL4R-IL21R*
16	rs982204	27399648	C	0.380	0.443	T	8.24E-08	0.77 (0.7-0.85)	imputed	intergenic	*IL4R-IL21R*
16	rs6498018	27400101	A	0.267	0.321	G	1.16E-06	0.77 (0.7-0.86)	imputed	intergenic	*IL4R-IL21R*
16	rs72535012	27403216	C	0.356	0.421	CTT	7.56E-08	0.76 (0.69-0.84)	imputed	intergenic	*IL4R-IL21R*
16	rs1116973	27405916	A	0.356	0.422	T	7.21E-08	0.76 (0.69-0.84)	imputed	intergenic	*IL4R-IL21R*
16	rs2382582	27406971	C	0.356	0.422	A	7.21E-08	0.76 (0.69-0.84)	imputed	intergenic	*IL4R-IL21R*
16	rs2189521	27413566	C	0.242	0.306	T	5.98E-09	0.73 (0.65-0.81)	genotyped	5'UTR	*IL21R*
19	rs350146	931523	T	0.519	0.459	C	5.05E-07	1.27 (1.16-1.4)	genotyped	intronic	*ARID3A*
19	rs10402860	939279	T	0.391	0.454	C	1.53E-07	0.77 (0.7-0.85)	imputed	intronic	*ARID3A*
19	rs10414193	939697	G	0.403	0.470	A	1.60E-08	0.76 (0.69-0.84)	genotyped	intronic	*ARID3A*
19	rs2283589	940400	A	0.227	0.288	G	1.42E-08	0.72 (0.65-0.81)	imputed	intronic	*ARID3A*
19	rs2283590	940674	T	0.227	0.288	A	1.42E-08	0.72 (0.65-0.81)	imputed	intronic	*ARID3A*

19	rs57616273	941325	C	0.220	0.277	T	6.81E-08	0.74 (0.66-0.82)	imputed	intronic	*ARID3A*
19	rs60224169	941423	T	0.396	0.464	C	2.53E-08	0.76 (0.69-0.84)	imputed	intronic	*ARID3A*
19	rs10415976	941603	G	0.414	0.486	A	1.05E-09	0.75 (0.68-0.82)	genotyped	intronic	*ARID3A*
19	rs62132348	941731	T	0.220	0.277	C	6.81E-08	0.74 (0.66-0.82)	imputed	intronic	*ARID3A*
19	rs201406961	941824	G	0.387	0.455	GCGTA	4.37E-08	0.76 (0.68-0.84)	imputed	intronic	*ARID3A*
19	rs72454678	941825	C	0.397	0.464	CGTAT	3.45E-08	0.76 (0.69-0.84)	imputed	intronic	*ARID3A*
19	rs10411830	941987	A	0.396	0.464	G	2.53E-08	0.76 (0.69-0.84)	imputed	intronic	*ARID3A*
19	rs35334454	942181	G	0.221	0.277	C	1.10E-07	0.74 (0.66-0.83)	imputed	intronic	*ARID3A*
19	rs7258301	942660	G	0.416	0.489	A	1.11E-09	0.74 (0.68-0.82)	imputed	intronic	*ARID3A*
19	rs2238573	945013	A	0.223	0.280	G	8.49E-08	0.74 (0.66-0.83)	genotyped	intronic	*ARID3A*
19	rs2238575	945426	A	0.223	0.280	G	8.78E-08	0.74 (0.66-0.83)	imputed	intronic	*ARID3A*

* Sequence position was annotated based on the GRCh37/hg19 assembly. Chr., chromosome; MAF, minor allele frequency. # P value is based on additive model without correction; OR, odds ratio, is calculated for minor allele

Supplementary Table 8: Comparison of independent associations in IL12A locus between Caucasian and Han PBC cohorts

	Caucasian Cohort* (2,861 cases-8,514 controls)						Han Chinese cohort (1122 cases-4036 controls)					
Signals	Lead SNP	RA	RAF	*P* value	LD region	LD size (bp)	Lead SNP	RA	RAF	*P* value#	LD region	LD size (bp)
1	rs2366643	A	0.57	3.92×10^{-22}	159,720,271-159,736,572	16,301	rs574808	A	0.82	3.70×10^{-3}	159,728,535-159,737,535	8,822
2	rs62270414	G	0.15	5.74×10^{-17}	159,630,577-159,692,282	51,705	no variant					
3	rs668998	G	0.43	4.73×10^{-9}	159,707,519-159,715,551	8,032	rs582537	A	0.77	6.55×10^{-8}	159,707,519-159,715,551	8,032
4	rs80014155	A	0.004	2.64×10^{-11}	159,625,393-159,694,053	68,660	no variant					

The significant loci found in IL12A locus. RA, risk allele; RAF, risl allele in controls; LD,linkage disequilibrium. * Results for Caucasian cohort was based on PBC Immunochip data from Liu JZ., et al., Nat Genet. 44(10):1137-41, 2012. # P value was based on the resuls in GWAS discovery stage in a 1022 PBC and 4036 control cohort.

Supplementary Table 9: Conditional analysis of rs925550 and rs17005934 in IL21 locus based on GWA samples

Test SNP	Conditioning SNP	GWAS (1122 cases / 4036 controls)			
		Unconditioned		Conditioned	
		OR	*P*	OR	*P*
rs925550	rs925550	1.27	6.21E-07	NA	NA
rs17005934		1.27	1.42E-06	1.21	5.93E-05
rs925550	rs17005934	1.27	6.21E-07	1.16	0.00145
rs17005934		1.27	1.42E-06	NA	NA

Supplementary Table 10: Expression quantitative loci (eQTLs) at novel PBC risk loci

Locus	SNP	LD (r^2)	Gene	Score	Tissue	Source (sample size)
16p12.1	rs2189521	Index	*IL21R*	4.00	PBMC	Westra, et al., 2013 (N=5311)
16p12.1	rs10852316	Index	*IL21R*	3.74	PBMC	Westra, et al., 2013 (N=5311)
16p12.1	rs722516	1	*IL21R*	3.72	PBMC	Westra, et al., 2013 (N=5311)
16p12.1	rs1107788	1	*IL21R*	3.71	PBMC	Westra, et al., 2013 (N=5311)
16p12.1	rs1859309	1	*IL21R*	3.71	PBMC	Westra, et al., 2013 (N=5311)
16p12.1	rs1859306	1	*IL21R*	3.71	PBMC	Westra, et al., 2013 (N=5311)
16p12.1	rs11074855	1	*IL21R*	3.71	PBMC	Westra, et al., 2013 (N=5311)
16p12.1	rs982204	0.98	*IL21R*	3.73	PBMC	Westra, et al., 2013 (N=5311)
16p12.1	rs1116973	0.98	*IL21R*	3.75	PBMC	Westra, et al., 2013 (N=5311)
1p13.1	rs2300747	Index	*CD58*	6.60	Lymphoblastoid cells	Stranger et al., 2007 (N = 210)
1p13.1	rs3850814	0.87	*CD58*	8.60	Lymphoblastoid cells	Stranger et al., 2007 (N = 210)
1p13.1	rs10754444	0.94	*CD58*	6.76	Lymphoblastoid cells	Stranger et al., 2007 (N = 210)
1p13.1	rs10924106	0.92	*CD58*	8.77	Lymphoblastoid cells	Stranger et al., 2007 (N = 210)
1p13.1	rs12031061	0.93	*CD58*	7.99	Lymphoblastoid cells	Stranger et al., 2007 (N = 210)
1p13.1	rs10924108	0.92	*CD58*	5.69	Lymphoblastoid cells	Stranger et al., 2007 (N = 210)
1p13.1	rs1414275	0.91	*CD58*	7.99	Lymphoblastoid cells	Stranger et al., 2007 (N = 210)
1p13.1	rs11588376	0.91	*CD58*	7.99	Lymphoblastoid cells	Stranger et al., 2007 (N = 210)
1p13.1	rs1016140	0.91	*CD58*	7.99	Lymphoblastoid cells	Stranger et al., 2007 (N = 210)
1p13.1	rs758518	0.93	*CD58*	7.99	Lymphoblastoid cells	Stranger et al., 2007 (N = 210)
1p13.1	rs12141411	0.88	*CD58*	7.99	Lymphoblastoid cells	Stranger et al., 2007 (N = 210)
1p13.1	rs12044852	0.91	*CD58*	7.66	Lymphoblastoid cells	Stranger et al., 2007 (N = 210)
1p13.1	rs1335532	0.98	*CD58*	7.02	Lymphoblastoid cells	Stranger et al., 2007 (N = 210)
15q25.1	rs11857713	Index	*IL16*	54.05	Monocyte	Zeller et al., 2010 (N = 1490)
15q25.1	rs4778636	Index	*IL16*	59.63	Monocyte	Zeller et al., 2010 (N = 1490)
15q25.1	rs4577037	Index	*IL16*	56.56	Monocyte	Zeller et al., 2010 (N = 1490)
15q25.1	rs4778889	0.79	*IL16*	14.07	Monocyte	Zeller et al., 2010 (N = 1490)
15q25.1	rs4778639	0.98	*IL16*	59.04	Monocyte	Zeller et al., 2010 (N = 1490)
19p13.3	rs10415976	Index	*ARID3A*		PBMC	Westra, et al., 2013 (N=5311)
19p13.3	rs7258301	1	*ARID3A*	3.45	PBMC	Westra, et al., 2013 (N=5311)
19p13.3	rs2238574	0.97	*ARID3A*	3.07	PBMC	Westra, et al., 2013 (N=5311)
19p13.3	rs4806860	0.95	*ARID3A*	3.07	PBMC	Westra, et al., 2013 (N=5311)
19p13.3	rs2238577	0.8	*ARID3A*	3.68	PBMC	Westra, et al., 2013 (N=5311)
19p13.3	rs2238573	Index	*ARID3A*	3.82	PBMC	Westra, et al., 2013 (N=5311)
19p13.3	rs12611059	0.84	*ARID3A*	3.38	PBMC	Westra, et al., 2013 (N=5311)
19p13.3	rs349306	0.72	*ARID3A*	4.36	PBMC	Westra, et al., 2013 (N=5311)

Candidate variants that are eQTLs in different tissues were identified using the searchable eQTL browsers and databases, seeQTL (http://www.bios.unc.edu/research/genomic_software/seeQTL/); Blood eQTL (http://www.genenetwork.nl/bloodeqtlbrowser/). 'Score' refers to the -log10P, a measure of the strength of association between the SNP and gene expression. The sources of eQTL data are listed in the reference [13-15].

Supplementary Table 11: Expression quantitative loci (eQTLs) at novel PBC risk loci

HaploReg_query_SNP	Variant rs ID	LD r2 (Asian)	LD D' (Asian)	chr	pos_hg38	RefSeq_name	dbSNP_functional_annotation	Ref	Alt	AFR	AMR	ASN	EUR	GERP_cons	SiPhy_cons	Motifs	RegulomeDB variant category	note
rs2300747	rs11588376	0.88	0.98	1	116529902	*CD58*	INT	T	C	0.34	0.22	0.58	0.12	0	0	Maf, Nrf1	1d	eQTL + TF binding + any motif + DNase peak
rs11556218	rs4778889	0.83	1	15	81296654	*IL16*	INT	T	C	0.42	0.23	0.22	0.16	0	0	Sox,TAL1	1f	eQTL + TF binding/DNase peak
rs11556218	rs4778891	1	1	15	81297489	*IL16*	INT	C	G	0.22	0.14	0.19	0.05	0	0	Mef2	1b	eQTL + TF binding + any motif + DNase footprint + DNase peak
rs11556218	rs11857713	1	1	15	81298924	*IL16*	INT	C	T	0.27	0.15	0.19	0.05	0	0	CTCF,ERalpha-a,Hoxb3,Nr2f2,RAR	1f	eQTL+TF binding/DNAse peak
rs11556218	rs4778636	1	1	15	81299298	*IL16*	INT	G	A	0.26	0.15	0.19	0.07	0	0		1f	eQTL+TF binding/DNAse peak
rs11556218	rs17875523	1	1	15	81304211	*IL16*	INT	C	T	0.2	0.14	0.19	0.07	0	0	BCL,Elf3,FEV,GATA, HMGN3,Hdx,Mef2,STAT,p300	2b	TF binding + any motif + DNase footprint + DNase peak
rs11556218	rs3898677	0.83	1	15	81304221	*IL16*	INT	T	C	0.49	0.23	0.22	0.16	0	0		1b	eQTL + TF binding + any motif + DNase footprint + DNase peak
rs11556218	rs4577037	1	1	15	81304319	*IL16*	INT	T	G	0.2	0.14	0.19	0.07	0	0	Foxq1,Nkx2	1b	eQTL + TF binding + any motif + DNase footprint + DNase peak
rs11556218	rs1803275	1	1	15	81306075	*IL16*	SYN	G	A	0.24	0.14	0.19	0.07	0	0	Maf	1f	eQTL + TF binding/DNase peak
rs11556218	rs3926279	0.99	1	15	81307102	*IL16*	INT	A	G	0.29	0.15	0.19	0.07	0	0	RORalpha1	1f	eQTL + TF binding/DNase peak
rs11556218	rs4778639	0.94	1	15	81308110	*IL16*	INT	T	G	0.02	0.11	0.18	0.06	0	0	GATA,GR	1f	eQTL + TF binding/DNase peak
rs11556218	rs4778640	0.9	0.99	15	81311790	*IL16*	U3	A	G	0.33	0.14	0.2	0.03	0	0	Pbx-1	1f	eQTL + TF binding/DNase peak
rs2189521	rs2107357	0.82	0.93	16	27399508	*IL21R*	Prom	A	G	0.81	0.85	0.71	0.89	0	0	Pax-4	1f	eQTL + TF binding/DNase peak
rs2238573	rs62132348	0.93	0.96	19	941731	*ARID3A*	INT	C	T	0.07	0.04	0.26	0.07	0	0	E2A, TBX5, ZEB1	1f	eQTL + TF binding/DNase peak
rs2238573	rs2238575	1	1	19	945426	*ARID3A*	INT	G	A	0.08	0.12	0.26	0.11	0	0	CHD2, EBF, NRSF, RXRA	2b	TF binding + any motif + DNase footprint + DNase peak
rs10415976	rs10414193	0.86	0.98	19	939697	*ARID3A*	INT	A	G	0.25	0.23	0.45	0.12	0	0	CTCF, E2A, Myc, Myf, Rad21, SMC3, TCF12	1b	eQTL + TF binding + any motif + DNase footprint + DNase peak
rs10415976	rs10404331	0.96	0.99	19	940927	*ARID3A*	INT	G	T	0.38	0.24	0.47	0.13	1	0	AhR, Egr-1, Hic1, Pax-4, RREB-1, RREB-1, Zfp281	2b	TF binding + any motif + DNase footprint + DNase peak
rs10415976	rs10404332	0.92	0.98	19	940928	*ARID3A*	INT	G	C	0.38	0.24	0.47	0.14	1	0	AP-1, Egr-1, HEY1, Hic1, RREB-1, Rad21, Smad4, TLX1, ZBTB33, Zfp281	2b	TF binding + any motif + DNase footprint + DNase peak
rs10415976	rs10415976	1	1	19	941603	*ARID3A*	INT	A	G	0.33	0.14	0.47	0.1	0	0	Pax-4, SREBP	2b	TF binding + any motif + DNase footprint + DNase peak
rs10415976	rs2238574	0.94	0.97	19	945089	*ARID3A*	INT	C	A	0.39	0.32	0.47	0.18	0	0	ERalpha-a, Esr2, HNF4, LRH1, ZBTB7A, ZBTB7A	2b	TF binding + any motif + DNase footprint + DNase peak

Supplementary Table 12: Comparison of PBC GWAS significant loci among three ethnic groups

		Caucasian GWAS meta analysis* (2,764 PBC and 10,475 control)			Japanese GWAS analysis# (1,274 PBC and 1,091 control)			Han Chinese GWAS analysis (2,029 PBC and 6,163 control)		
**Locus**	**Candidate Genes**	**Lead SNP**	***P***	**OR**	**Lead SNP**	***P***	**OR**	**Lead SNP**	***P***	**OR**
1p13.1	*CD58*							rs2300747	1.84×10^{-12}	1.29
1p31.3	*IL12RB2*， *SERBP1*	rs6679356	7.49×10^{-28}	1.52						
1q31.3	*DENND1B*	rs17641524	1.01×10^{-11}	0.77						
2p23.1	*LBH**	rs4952108	5.05×10^{-8}	1.28						
2q32.3	*NAB1*， *STAT1*， *STAT4*	rs3771317	2.32×10^{-14}	0.71	rs7574865	1.11×10^{-6}	1.35	rs10168266	3.95×10^{-13}	1.31
2q33.2	*CD28, CTLA4*							rs7599230	3.30×10^{-10}	1.31
3p24.3	*PLCL2*	rs1372072	3.99×10^{-8}	1.2						
3q13.33	*TMEM39A*， *CD80*	rs2293370	4.26×10^{-15}	1.42				rs3732421	3.10×10^{-13}	0.74
3q25.33	*IL12A*， *SCHIP1*	rs485499	1.43×10^{-23}	0.71				rs582537	2.36×10^{-11}	0.75
4q24	*MANBA*， *NFKB1*	rs1054037	8.31×10^{-10}	1.22				rs1598856	1.80×10^{-10}	1.26
4q27	*IL21*							rs925550	3.87×10^{-13}	1.31
5p13.2	*IL7R*， *CASPL*	rs860413	4.73×10^{-11}	1.28	rs6890853	3.66×10^{-8}	1.47			
6p21.3	Many (MHC)	rs7774434	2.37×10^{-56}	1.68				rs9268644	7.83×10^{-31}	0.51
7q32.1	*IRF5, TNP03*	rs10488631	5.08×10^{-23}	0.63						
9q32	*TNFSF15,TNFSF8*				rs4979464	2.84×10^{-14}	1.56	rs4979467	1.22×10^{-29}	1.53
11q13.1	*CCDC88B,* others	rs510372	1.68×10^{-6}	0.81						
11q23.1	*POU2AF1*				rs4938534	2.38×10^{-8}	1.39			
11q23.3	*CXCR5, DDX6*	rs6421571	1.78×10^{-13}	1.39				rs77871618	2.56×10^{-13}	1.4
12p13.31	*TNFRSF1A, PLEKHG6*	rs1800693	1.84×10^{-9}	0.82				rs4149576	3.81×10^{-9}	1.37
12q24.12	*ATXN2, SH2B3, TRAFD1, ALDH2*	rs11065987	3.20×10^{-8}	0.84						
13q14.3	*DLEU1**	rs9591325	1.07×10^{-10}	1.63						
14q24.1	*RAD51L1, RAD51B*	rs911263	2.25×10^{-9}	1.24						
14q32.32	*EXOC3L4, TNFAIP2*	rs2297067	6.34×10^{-19}	0.72						
15q25.1	*IL16*							rs11556218	8.99×10^{-9}	1.29
16p12.1	*IL4R, IL21R*							rs2189521	4.00×10^{-16}	0.71
16p13.13	*SOCS1, CLEC16A, DEXI*	rs12924729	2.39×10^{-14}	1.31						
17q12	*IKZF3,* others	rs9303277	2.57×10^{-11}	0.81	rs9303277	3.66×10^{-9}	1.44	rs9635726	1.62×10^{-16}	1.37
19p13.2	*TYK2*	rs2304256	1.05×10^{-10}	0.79						
19p13.3	*ARID3A*							rs10414193	5.80×10^{-11}	0.77
19q13.3	*SPIB, MYBPC2*	rs3745516	1.22×10^{-20}	0.72						
22q13.1	*SYNGR1, PDGFB*	rs2069235	2.23×10^{-11}	0.79				rs137603	2.67×10^{-8}	0.73

The loci defined as GWAS significance based on *P* value exceeding 5 ×10-8 identified in published studies or in this manuscript. * Caucasian GWAS results were based on the publication by Cordell, AJ. et al.,(ref. 5). #Japanese GWAS data were based on the publication by Nakamura, M. et al.,(ref. 8). OR, odds ratio, is calculated for minor allele.

Supplementary Table 13: Loci achieved GWA significance (P <5×10-8) in this study overlapping with other autoimmune diseases

Chr	SNP	Position* (bp)	Genes in the Region	Combined P Value	Overlap Loci with other Autoimmune Diseases	Reference (PMID)
6p21	rs9268644	32408044	Many (MHC)	7.83×10^{-31}	Many	
9q32	rs4979467	117630043	*TNFSF15,TNFSF8*	1.22×10^{-29}	CD, UC	16221758, 21297633
17q12	rs9635726	38020141	*IKZF3*, others	1.62×10^{-16}	CD, RA, MS, T1D, UC	23143596, 24387989,
16p12.1	rs2189521	27413566	*IL4R, IL21R*	4.00×10^{-16}		current study
2q33.2	rs4675369	204643194	*CD28, CTLA4*	1.38×10^{-13}	GD, T1D, AA, RA, CeD, MS, PSC	21841780, 19430480, 20596022, 20453842, 20190752, 26475045, 23603763
11q23.3	rs77871618	118733624	*DDX6, CXCR5*	2.56×10^{-13}	vitiligo, RA, MS	22951725, 21383967, 24387989
3q13.33	rs3732421	119150089	*CD80*	3.10×10^{-13}	vitiligo, SLE, CeD	22951725, 23273568, 20190752
4q27	rs925550	123588526	*IL21*	3.87×10^{-13}	AA, CeD, UC, T1D, AD, PSC, JIA, RA	20596022, 17558408, 21297633, 23727859, 23603763, 23603761, 23143596
2q32.3	rs10168266	191935804	*STAT1,STAT4*	3.95×10^{-13}	SjS, SLE, BD, SSc, MS, JIA, RA	20383147, 19838193, 19333953, 18516230, 23291587, 24387989, 23603761, 23143596
1p13.1	rs2300747	117104215	*CD58*	1.84×10^{-12}	MS,	21833088
19p13.3	rs10415976	941603	*ARID3A*	3.61×10^{-12}		current study
3q25.33	rs582537	159710098	*IL12A, SCHIP1*	2.36×10^{-11}	CeD, MS, BD, SSc	18311140, 20555355, 27548383, 24387989
4q24	rs1598856	103446115	*NF-kB1*	1.80×10^{-10}	MS, Pso, Sarcoidosis	21833088, 25006012, 26051272
12p13.31	rs4149576	6449115	*TNFRSF1A*	3.81×10^{-9}	MS,AS	19525953, 23749187
15q25.1	rs11556218	81598269	*IL16*	8.99×10^{-9}		current study
16q21	rs2550374	58254448	*CSNK2A2, CCDC113*	1.51×10^{-8}		current study
22q13.1	rs137603	39694225	*RPL3,SYNGR1, PDGFB*	2.67×10^{-8}	UC, RA, CD	24532676,

The loci defined as overlaping based on P value exceeding 5 X 10-8 identified in published studies. References cited in this table were listed with Pubmed ID (PMID). Chr, chromosome; SNP, single nucleotide polymorphism; AA, Alopecia areata; AD,atopic dermatitis; AS, ankylosing spondylitis; BD, Behçet's disease; CD, Crohn disease; CeD, celiac disease; GD, Graves' disease; JIA, juvenile inflammatory arthritis; MS, multiple sclerosis; PSC, primary sclerosing cholangitis; Pso, psoriasis; RA, rheumatoid arthritis; SLE, systemic lupus erythematosus; SjS, Sjogren syndrome; SSc, systemic sclerosis; T1D, type 1 diabetes mellitus; UC, ulcerative colitis.
